# Enhanced Methanol Synthesis from CO_2_ Hydrogenation Achieved by Tuning the Cu–ZnO Interaction in ZnO/Cu_2_O Nanocube Catalysts Supported on ZrO_2_ and SiO_2_

**DOI:** 10.1021/jacs.4c01077

**Published:** 2024-03-12

**Authors:** David Kordus, Simon Widrinna, Janis Timoshenko, Mauricio Lopez Luna, Clara Rettenmaier, See Wee Chee, Eduardo Ortega, Osman Karslioglu, Stefanie Kühl, Beatriz Roldan Cuenya

**Affiliations:** † Department of Physics, Ruhr-University Bochum, 44780 Bochum, Germany; ‡ Department of Interface Science, Fritz-Haber Institute of the Max Planck Society, 14195 Berlin, Germany

## Abstract

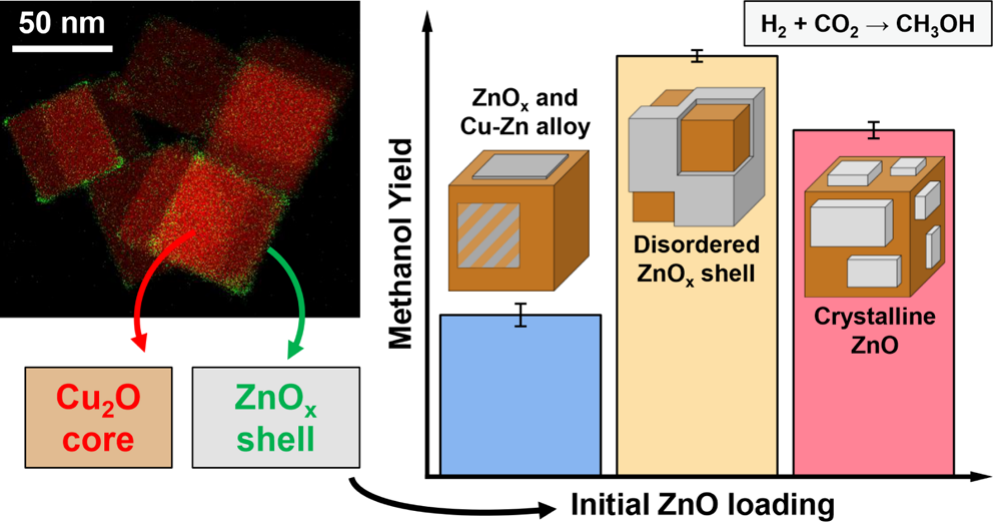

The nature of the Cu–Zn interaction and especially the role of Zn in Cu/ZnO catalysts used for methanol synthesis from CO_2_ hydrogenation are still debated. Migration of Zn onto the Cu surface during reaction results in a Cu–ZnO interface, which is crucial for the catalytic activity. However, whether a Cu–Zn alloy or a Cu–ZnO structure is formed and the transformation of this interface under working conditions demand further investigation. Here, ZnO/Cu_2_O core–shell cubic nanoparticles with various ZnO shell thicknesses, supported on SiO_2_ or ZrO_2_ were prepared to create an intimate contact between Cu and ZnO. The evolution of the catalyst’s structure and composition during and after the CO_2_ hydrogenation reaction were investigated by means of *operando* spectroscopy, diffraction, and *ex situ* microscopy methods. The Zn loading has a direct effect on the oxidation state of Zn, which, in turn, affects the catalytic performance. High Zn loadings, resulting in a stable ZnO catalyst shell, lead to increased methanol production when compared to Zn-free particles. Low Zn loadings, in contrast, leading to the presence of metallic Zn species during reaction, showed no significant improvement over the bare Cu particles. Therefore, our work highlights that there is a minimum content of Zn (or optimum ZnO shell thickness) needed to activate the Cu catalyst. Furthermore, in order to minimize catalyst deactivation, the Zn species must be present as ZnO_*x*_ and not metallic Zn or Cu–Zn alloy, which is undesirably formed during the reaction when the precatalyst ZnO overlayer is too thin.

## Introduction

Methanol is an important chemical with diverse applications as a precursor to the synthesis of other chemicals or directly as a fuel.^1^ Therefore, methanol synthesis is an established industrial process used for many decades. With the ongoing advance of climate change, methanol synthesis using green hydrogen from electrolysis has become very attractive, especially when combined with a carbon capture technology enabling to recycle CO_2_ and thus to reduce the net amount of anthropogenic CO_2_ emissions.^2^

The industrial methanol synthesis process uses a Cu/ZnO/Al_2_O_3_ catalyst, where Cu and ZnO form the active site.^1,3,4^ While ZnO alone is not active for methanol synthesis,^5,6^ in combination with Cu, it greatly enhances the catalyst’s activity, but the mechanisms behind are still debated. Therefore, tracking the evolution of the Cu–ZnO structure under reaction conditions is crucial to understand the most active phases of the catalyst. Multiple studies showed the migration of Zn from the surrounding ZnO onto the Cu particles during the reaction in Cu/ZnO catalysts,^7−10^ leading to an intimate Cu–Zn contact. This creates a complex interface leading to the synergetic effect of Cu and ZnO. Additionally, it has been proposed that Zn could provide a reservoir of atomic hydrogen, promoting hydrogen spillover^11^ or that the ZnO support induces lattice strain and defects in Cu that acted as active material.^12^

Nonetheless, despite excellent prior work, a subject of ongoing debate is whether during reaction Zn can be found as ZnO,^13,14^ as partially oxidized Zn^δ+^ or ionic Zn species,^15−18^ or as a Cu–Zn alloy.^3,19^ Depending on the reaction conditions, there can also be mixtures of different chemical states and structures^18−20^ so that one has yet to determine which state actually participates in the formation of the active sites. As for the alloy formation, it was debated whether its presence is beneficial^3,5,21,22^ or unfavorable^14,23^ for methanol synthesis. An important matter in this context is whether the possible Cu–Zn alloy even forms under the conditions relevant for the reaction, i.e., at the elevated pressures (around 50 bar) and temperatures (200–250 °C) which are typical for the industrial process, but might not be achievable with most characterization techniques commonly employed.^4,24−27^ The alloy formation might only take place under certain conditions, especially at high temperatures,^10,21,28^ and might not be stable under other reaction conditions,^29,30^ especially when significant amounts of water are produced during CO_2_ hydrogenation.^31^ Nonetheless, a recent *operando* spectroscopic work from our group clearly revealed catalyst deactivation upon Cu–Zn alloy formation on CuZn NPs supported on SiO_2_.^32^ In addition, a model has also been proposed where Zn migrates onto Cu and transforms into ZnO layers or particles with time under reaction conditions,^30^ suggesting that the Cu–Zn alloy might only be present initially, but disappears after extended time under reaction conditions.

Apart from the oxidation state of Zn, it was also shown that the Zn coverage or the amount of Zn in the catalysts plays a crucial role^5,10,26,27,33^ for the activity and that there might be an optimal coverage, although the exact values for this differ.

To summarize, it can be concluded that the Cu–ZnO interaction, depending on the individual reaction conditions and initial structure of the precatalyst, can result in a multitude of different structures of the working catalyst under reaction conditions, which cannot always be easily determined. This, however, is critical for the correct determination of the active sites, especially if this information has to be transferred to a theoretical model that depends on a correct material assumption under the reactive environment for the calculations.

The goal of this work is to closely look at the role of the Cu–ZnO interaction for the methanol synthesis activity by creating a model precatalyst material where the ZnO is already “segregated”, i.e., available on the surface of Cu, initially as ZnO, so that the contact area of Zn and Cu is maximized. This has been achieved by synthesizing shape-controlled cubic Cu_2_O precatalysts overcoated by ZnO layers of different thicknesses. In this way, Cu and ZnO are in close contact from the start of the reaction, and a migration of Zn from a bulk ZnO support to form an intimate contact between the two components is not needed. Thereby, the different effects from a well-controlled amount of Zn can be explored on a working catalyst under the relevant reaction conditions. Moreover, in this system, we can not only explore the role of the ZnO/Cu interface but also that of the functional interface with the underlying ZrO_2_ or SiO_2_ support.

## Experimental Section

### Catalyst Preparation

The synthesis of Zn-free Cu_2_O NCs was adapted from previously reported procedures.^34,35^ The base chemicals used for the production of the solutions that are used during the synthesis are l-ascorbic acid (Sigma-Aldrich, ≥98%), NaOH (Fischer scientific, ≥97%), CuSO_4_·5H_2_O (Sigma-Aldrich, 99.995%), and ZnCl_2_ (Sigma-Aldrich, 99.999%). 457.5 mL of ultrapure (18.2 MΩ·cm) water was mixed with 5 mL of CuSO_4_ (0.1 M) in a conical flask under constant stirring. Then, 17.5 mL of NaOH (1.0 M) and 20 mL of l-ascorbic acid (0.25 M) are added. This solution is kept stirring for 15 min.

In order to deposit a Zn shell on the cubes, the previous procedure was followed by the addition of ascorbic acid. About 12.5 min after the initial addition of the ascorbic acid, ZnCl_2_ (0.1 M) and additional ascorbic acid (0.25 M) were added to the solution. The amounts of ZnCl_2_ and ascorbic acid added determine the thickness of the Zn shell. For the low Zn loading, 1.67 mL of ZnCl_2_ and 3.33 mL of l-ascorbic acid were added; for the medium Zn loading, 5 mL of ZnCl_2_ and 10 mL of l-ascorbic acid were added; and for the high Zn loading, 15 mL of ZnCl_2_ and 15 mL of l-ascorbic acid were added. The solutions were then stirred for an additional minute. After the synthesis, the obtained solution is immediately centrifuged (15 min, 4600 rpm); afterward, the supernatant is discarded, and the precipitate is washed with a 1:1 mixture of water and ethanol. The centrifugation and washing process is repeated four times. The resulting particles are stored in ethanol.

For the preparation of the final catalyst, the ZnO/Cu_2_O core–shell nanocubes were mixed with an oxide support. For all purposes, Cu_2_O and ZnO/Cu_2_O NCs were supported on ZrO_2_ (Sigma-Aldrich). For some additional reference X-ray absorption spectroscopy (XAS) measurements, a catalyst was prepared with the particles supported on SiO_2_ (Strem Chemicals). The particles were mixed with the oxide support in a 30:70 ratio by weight.

The final content of Zn on the Cu_2_O NCs can be adjusted by using different amounts of Zn during the synthesis. In total, a series of four different NC samples, differing only by the amount of ZnO, were prepared.

### Catalyst Characterization

#### Scanning Transmission Electron Microscopy (STEM)

STEM characterization was done with a Talos F200X (Thermo Fischer Scientific) microscope. Samples were prepared by using lacey carbon-coated gold transmission electron microscopy (TEM) grids. TEM characterization after reaction was done without exposure to air by transfer in a sealed reaction tube and sample preparation was carried out in an argon or N_2_-filled glovebox and subsequent transfer in a vacuum transfer holder. The entire procedure was described in detail previously.^32^ Additionally the STEM measurements were combined with energy-dispersive X-ray spectroscopy (EDX) to obtain elemental maps of the recorded images.

#### X-ray Diffraction (XRD)

XRD was acquired with a D8 Advance diffractometer (Bruker AXS) equipped with a Cu Kα source and a LynxEye XE-T detector. XRD patterns were recorded in a 2θ range of 20–90°, applying an increment of 0.005°. Rietveld refinement was performed using the software package TOPAS (Bruker AXS) to analyze the diffraction pattern.

For *in situ* XRD the samples were loaded in a reaction cell that is connected to a gas manifold consisting of multiple mass flow controllers. This way a consistent and accurate gas flow is achieved. Catalyst reduction was performed in 10% H_2_ in He at atmospheric pressure with a total flow rate of 100 mL/min and a temperature range from 100 to 250 °C. Measurements were performed in steps of 10 °C. After the reduction, the reaction gas mixture was introduced H_2_ + CO_2_ (3:1) and the cell pressurized to 10 bar. Measurements under reaction conditions were performed at 220, 250, and 400 °C. Diffractograms under reaction conditions were acquired after staying for 2 h at each condition. Additional remarks regarding the background during the *in situ* XRD measurements are given in Supplementary Note 1 together with Figure S1.

#### *Operando* X-ray Absorption Spectroscopy (XAS)

XAS spectra of ZnO/Cu_2_O/SiO_2_ catalysts were measured at beamline 2–2 at the Stanford Synchrotron Radiation Lightsource (SSRL). For the measurements, the samples were loaded in quartz capillaries (Hilgenberg GmbH) and mounted in a custom-made reactor cell connected to the gas dosing system, similar to previously described designs.^36^ Measurements were performed in fluorescence mode. Analysis of the outlet gas composition during the *operando* measurements was done by mass spectrometry. XAS data alignment, normalization, and linear combination fitting were done with the Athena software.^37^

Additional XAS measurements of ZnO/Cu_2_O/SiO_2_ and ZnO/Cu_2_O/ZrO_2_ catalysts were performed at the CLÆSS beamline of the ALBA synchrotron. For these measurements, a solid–gas reactor cell^38^ (ITQ) was used. Here, the samples were diluted with boron nitride for an optimal signal and pressed into pellets. Samples were measured in transmission or fluorescence mode, depending on the Zn loading on the sample. For low Zn loadings, the samples had to be measured in fluorescence to obtain optimum signal-to-noise ratios. More information regarding these additional measurements is given in Supplementary Note 2 and Figure S2.

#### X-ray Photoelectron Spectroscopy (XPS)

XPS was performed in a UHV system (SPECS) using a monochromatic Al Kα X-ray source (*h*v = 1486.6 eV) and a hemispherical analyzer (Phoibos 150). For the measurements, the powder samples were drop-cast on a Si(111) wafer. A flood gun (SPECS FG 15/40) was used to compensate sample charging. Spectra were aligned to the Si 2p_3/2_ peak at 99.4 eV. Processing of the XPS data was done with CasaXPS software.

#### Inductively Coupled Plasma–Mass Spectrometry (ICP-MS)

ICP-MS by an iCAP RQ (Thermo Scientific) was used to determine the elemental composition of the samples. Prior to ICP analysis, the samples were dissolved in a mixture of 6 mL of HCl, 2 mL of H_2_SO_4_, and 2 mL of HNO_3_ and subsequently digested in a microwave (Multiwave GO, Anton Paar) at 180 °C for 30 min. For the ICP measurement, the samples were further diluted with ultrapure water (18.2 MΩ·cm).

#### Catalytic Performance

Catalytic performance of the materials was investigated in a packed bed flow-reactor setup, and the reaction products were analyzed by online gas chromatography (GC) by a 7890B gas chromatograph (Agilent) equipped with a flame ionization detector (FID) and two thermal conductivity detectors (TCD). Typically, about 100 mg of catalyst was mixed with 600 mg SiC. Prior to reaction, the catalysts were first oxidized (synthetic air, 350 °C for 1 h). This pretreatment serves to remove all of the leftover carbon from the synthesis. Due to the ZnO shell, the carbon originating from the ascorbic acid used during the synthesis is harder to remove by washing the particles, which makes this pretreatment necessary. Subsequently, the catalysts were reduced (10% H_2_ balanced in He, 245 °C for 2 h). The catalytic activity was measured under methanol synthesis conditions using a gas mixture of 60% H_2_, 20% CO_2_, and 20% He (total flow: 50 mL/min, gas hourly space velocity (GHSV) = 6000 h^–1^), where the latter serves as an internal standard for the GC measurement. The catalyst was heated to 250 °C and examined in multiple steps with increasing pressure (10, 20, 40, and 60 bar). Each step lasted 12 h to ensure that the activity of the catalysts is measured under stable conditions.

## Results

### *Ex Situ* Structural and Compositional Characterization

Cu_2_O cubes with a ZnO shell are used as model catalysts for our studies. Four catalysts with different Zn loading (no Zn, low, medium, and high) were synthesized. The Cu/Zn ratios as measured with ICP-MS are shown in Table 1. Additionally, the Cu/Zn ratios were also estimated with the more surface-sensitive XPS method, and as expected, lower Cu/Zn ratios were obtained, due to Zn being at the very surface of the particles, Figure S3 and Supplementary Note 3. From the ICP and XPS measurements, we could also estimate the thickness of the ZnO layer on the Cu_2_O cubes. Details of the calculations can be found in Supplementary Note 4. Using the ICP data to calculate the layer thickness, we have estimated for the lowest Zn loading a ZnO thickness of 0.1 nm; for the medium Zn loading a thickness of 0.6 nm; and for the highest Zn loading a thickness of 2.6 nm. If we instead use the XPS data, the thicknesses are 0.05 nm for the low Zn loading catalyst, 0.3 nm for the medium Zn loading catalyst, and 1.0 nm for the high Zn loading catalyst. The discrepancy between the values obtained from the two different methods is due to the surface sensitivity of XPS and the nonhomogeneous nature of the ZnO overlayer on the nanocube’s surface. However, the combined analysis gives us a good estimate of the approximate average layer thickness.

**Table 1:** Cu/Zn Ratios for All As-Prepared Catalysts as Measured by ICP-MS and XPS

Zn content	Cu/Zn “bulk” ratio (ICP-MS)	Cu/Zn “surface” ratio (XPS)
no Zn	100/0	100/0
high	75.4/24.6	46.4/53.6
medium	93.8/6.2	78.9/21.1
low	98.9/1.1	96.2/3.8

The ZnO/Cu_2_O core–shell NCs were supported on a nanocrystalline ZrO_2_ powder to reduce the NC sintering during the reaction. It was shown that monoclinic ZrO_2_ in a Cu/ZrO_2_ catalyst is less active than the tetragonal form.^39^ XRD measurement of the support material (Figure S4) unveiled that the ZrO_2_ used is mostly in its more stable monoclinic form (99.5%), with only 0.5% tetragonal/cubic ZrO_2_. However, the Cu/ZrO_2_ and ZnO/ZrO_2_ interfaces in our catalyst may still contribute beneficially to the catalytic activity. It is reasonable to assume that the changes we observe between the activities of our catalysts originate mainly from the changed Cu/ZnO interface. The influence of ZrO_2_, due to its presence in its monoclinic form, should be comparatively small and also similar for all ZnO/Cu_2_O/ZrO_2_ catalysts (see also Supplementary Note 5 for more details).

**Figure 1:**
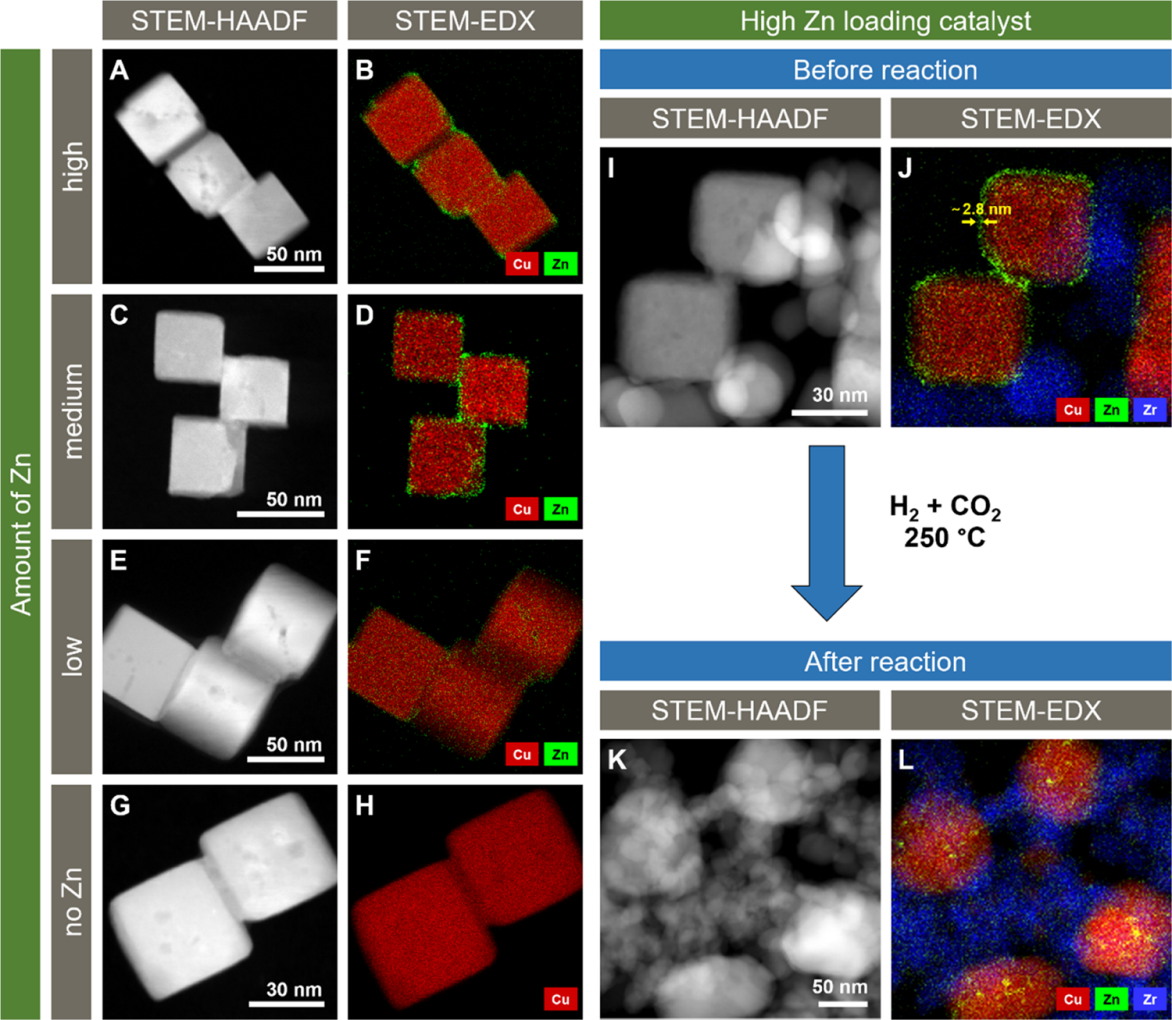
STEM images and corresponding EDX maps of unsupported Cu_2_O nanocubes with (A, B) high, (C, D) intermediate, or (E, F) low Zn loading and (G, H) without Zn. STEM images and EDX maps of the NCs with high Zn loading supported on ZrO_2_ are shown (I, J) before and (K, L) after reaction (CO_2_ + H_2_, 250 °C, 10, 20, 40, and 60 bar, 12 h at each condition). In the EDX maps, red represents Cu, green represents Zn, and blue represents Zr. The arrows in (J) roughly indicate the ZnO layer thickness on the Cu_2_O particles.

The unsupported Cu_2_O NCs (Cu_2_O cubes with the ZnO shell, but without the ZrO_2_ or SiO_2_ support) were measured with STEM. Exemplary STEM images and the corresponding EDX maps are shown in Figure. 1. The average particle size is 40–50 nm, with all samples having similar particle size distributions (Figure S5). By STEM–EDX it was confirmed that a core–shell structure with a Cu_2_O core and a ZnO shell is present and that the thickness of the shell could be varied by changing the Zn content during the synthesis. EDX spectra corresponding to the EDX maps of Figure 1A–H are shown in Figure S6. Moreover, for the highest Zn loading, it appears that Zn preferably attaches to the edges or corners of the Cu_2_O NCs. Therefore, the ZnO shell might not be arranged as a perfect layer but as an inhomogeneous layer made out of ZnO patches of variable thickness, potentially exposing some of the underlying Cu. However, estimations of the ZnO film thickness from EDX maps (e.g., approximately 2.8 nm as shown in Figure 1J) lead to similar thicknesses as calculated before from ICP measurements. Therefore, despite the not perfectly homogeneous distribution of ZnO, the calculated values seem to give a good approximation for the average ZnO layer thickness.

STEM images of the particles after reaction show that Zn is dispersed over the whole sample, revealing that ZnO partially migrates onto the ZrO_2_ support (Figures S7–S14). Additionally, we find that Zn, which is still mainly located on Cu, tends to form small particles. This particle formation is independent of the initial Zn loading. The cubic shape of the Cu particles is partially lost, which was also observed previously.^40^

### Reactivity

**Figure 2:**
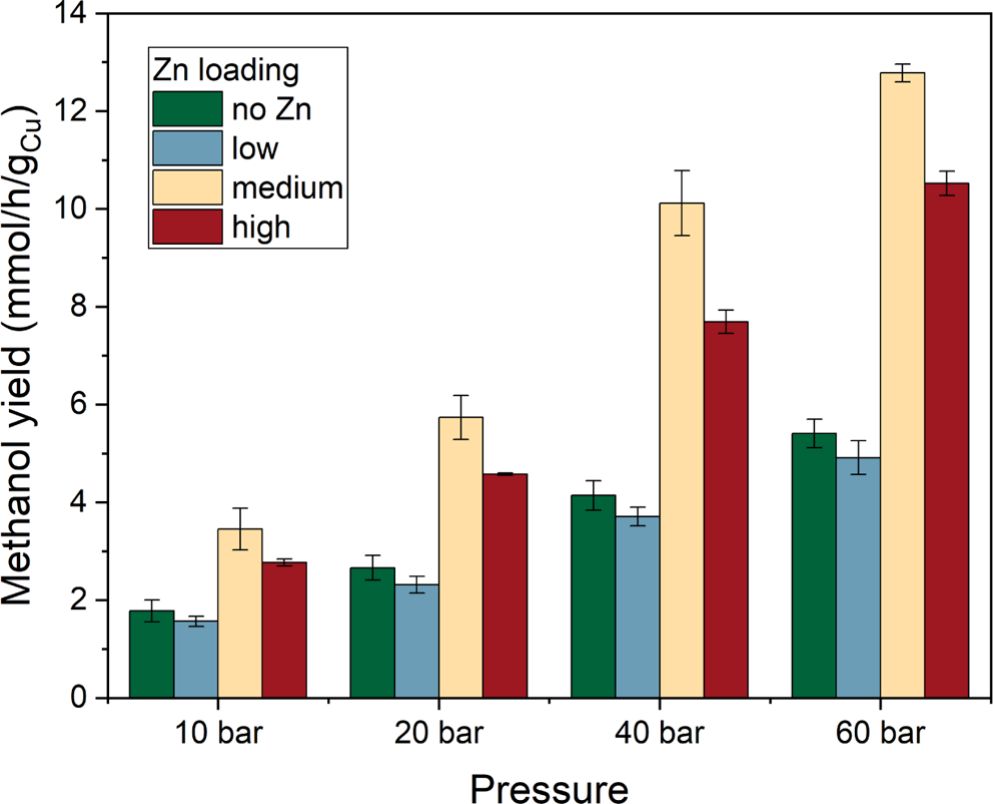
Methanol production of ZnO/Cu_2_O NC catalysts supported on ZrO_2_ with different Zn loadings. Measurements were performed under a H_2_ + CO_2_ + He (3:1:1) atmosphere at various pressures and at a temperature of 250 °C.

The catalytic performance of the ZnO/Cu-NC/ZrO_2_ samples was evaluated at a temperature of 250 °C and pressures of 10, 20, 40, and 60 bar. The results of these experiments are presented in Figures 2 and S15. As expected, the methanol yield increased with increasing pressure. The promotional effect of ZnO was only observed after a certain Zn surface coverage, with low Zn contents not resulting in an improvement in the methanol yield as compared to the pristine Cu_2_O NC precatalysts. Only when the amount of Zn was increased above at least 1.1% (ICP), a significant improvement is observed. The samples with medium and high Zn loadings were found to be better in terms of their catalytic activity. However, the catalyst with the medium Zn loading even outperforms the catalyst with the highest Zn loading, revealing that there is an optimum maximum Zn content, above which no further activity increase can be achieved. Thus, it appears that the relation between the Zn content on the Cu catalyst surface and the catalytic activity is not monotonous and that if a given Zn coverage on the Cu particles is exceeded, it is actually detrimental for the catalytic activity.^5,26^

### *In Situ* and *Operando* Characterization of the Catalyst Structural and Compositional Evolution (XRD, XAS)

**Figure 3:**
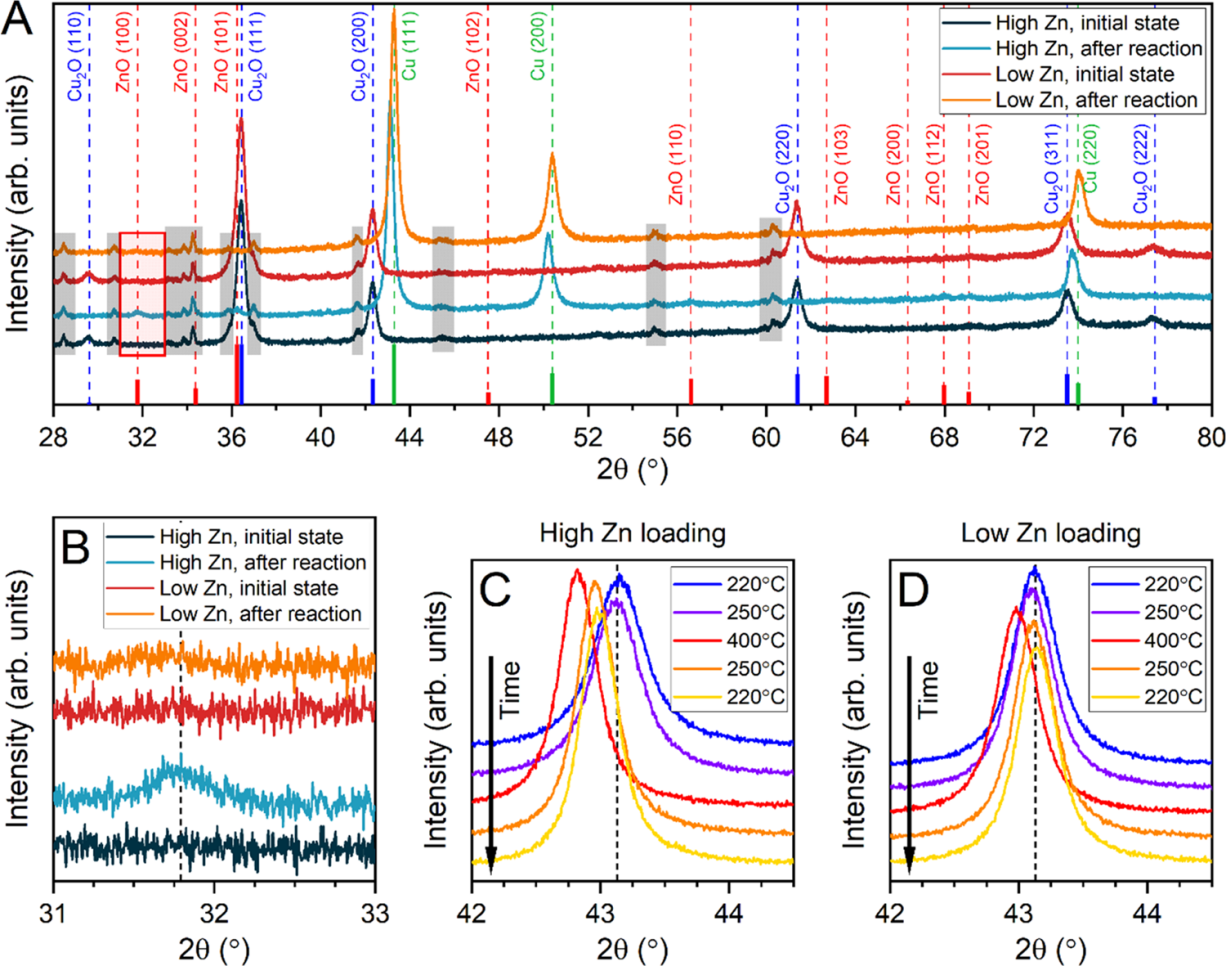
(A) XRD patterns of the unsupported ZnO/Cu catalysts with the highest and lowest Zn content in their initial state and after exposure to reaction conditions (H_2_ + CO_2_ (3:1), *p* = 10 bar). Peaks not assigned by the reference lines and marked with gray areas originate from the background of the sample holder. Contributions of ZnO can only be observed on the catalyst with the high Zn loading after reaction, as shown by the peaks marked with the red frame and in the zoomed-in region shown in (B). Shifts of the Cu(111) peaks due to exposure to different reaction temperatures are shown for the catalyst with (C) high and (D) low Zn loadings. Diffractograms were obtained in the chronologic order from top to bottom as indicated by the arrows so that the lines at the bottom in orange and yellow were measured after heating the catalyst to 400 °C. The scans that were performed *in situ* under reaction conditions were started after staying for 2 h at the respective conditions.

To get more information about the crystalline structure of Cu and Zn under reaction conditions, *in situ* XRD was used, as shown in Figure 3. Only the catalysts with the lowest and the highest Zn loadings were investigated with XRD. Furthermore, to avoid overlap with peaks from the support material, only the unsupported cubes with the Zn shell were measured. Also note that some peaks in the spectra originate from the sample holder background used for the *in situ* measurements and not from the sample itself (see also Supplementary Note 1).

In the initial state, only the peaks corresponding to Cu_2_O can be identified (Figure 3A). This is assigned to the cubic core of the particles. No peaks corresponding to either metallic Zn, ZnO, or any other crystalline Zn-containing compound can be observed. This unveils that Zn is initially present in the precatalyst as a highly disordered and noncrystalline form. Next, analogously to the reactivity measurements, the catalyst was reduced prior to the reaction (10% H_2_ balanced in He at 250 °C). While the temperature was increased, a diffractogram was recorded every 10 °C and a selection of the XRD patterns in the range of 140–190 °C is shown in Figure S16. From these measurements, the reduction of the Cu_2_O NCs to metallic Cu can be observed between the temperatures of 150 and 180 °C for both ZnO/Cu_2_O samples. This is similar to the previously investigated Cu_2_O NCs (without the ZnO shell) supported on bulk ZnO.^40^ The peaks corresponding to crystalline Cu_2_O completely disappear, and instead, metallic Cu features set in. A contribution including Zn cannot be identified in the XRD data measured *in situ* during the reduction treatment, which is due to the low content of Zn in these samples. Furthermore, Zn is only located in a thin shell on top of the cubes and therefore most likely not in a very crystalline state.

After reduction, the samples were exposed to the CO_2_ hydrogenation reaction conditions (75% H_2_ + 25% CO_2_ at 10 bar and 250 °C). No obvious changes in the chemical state could be observed from the spectra acquired under *in situ* conditions during this treatment. Afterward, a high-resolution scan of the sample was acquired at a lower temperature. Figure 3 shows a comparison of the spectra before and after the reaction. In the initial state, both samples are very similar, and only reflections belonging to Cu_2_O can be identified. After the reaction, Cu_2_O has completely disappeared and only metallic Cu remains. After the reaction with a high-resolution scan, a small Zn contribution (around 5%) was identified for the sample with a high Zn content (Figure 3B). Fitting parameters obtained with Rietveld refinement can be found in Table S2. A clear contribution of ZnO can only be identified in the catalyst with the highest Zn loading after the reaction, as revealed by the features at 31.8 and 36.2° corresponding to the (100) and (101) reflections of ZnO. For the sample with low Zn content, no peaks corresponding to any crystalline Zn compound could be identified, indicating that Zn is mainly still present in a highly disordered form for this catalyst or in amounts below the detection limit.

Furthermore, we wanted to look at the possible Cu–Zn alloy formation. Due to the high proportion of Cu, a Cu–Zn alloy would most likely also display a fcc structure with lattice parameters close to that of metallic Cu (α-Brass).^41,42^ The incorporation of Zn into the Cu lattice would lead to a small increase of the lattice parameter following Vegard’s law.^43^ This increase is indeed observed for Cu/ZnO catalysts that are exposed to high temperatures (>300 °C), where the formation of brass is well known.^14^ In our catalysts, the identification of brass via XRD is difficult because of the low content of Zn, and its highly disordered nature, which would at best consequently lead to only a small fraction of brass overlapping with the much bigger contribution from metallic Cu. Nevertheless, we exposed the catalysts to three different reaction temperatures (220, 250, and 400 °C) to force the alloy formation. Indeed, we observed a shift in our Cu peaks (Figure 3C,D) induced by the Cu–Zn alloy formation. The corresponding lattice parameters obtained with Rietveld refinement are given in Table S3. For the catalyst with the high Zn loading, at 220 °C (*a* = 3.631 Å) and 250 °C (*a* = 3.634 Å), the first peak shift observed in Figure 3C can be explained well by thermal expansion only. However, further heating the catalyst to 400 °C (*a* = 3.661 Å) leads to irreversible alloy formation,^18^ indicated by the fact that the lattice parameters are higher than before after going back to 220 °C (*a* = 3.646 Å) and 250 °C (*a* = 3.647 Å). For the catalyst with the low Zn loading, it is harder to detect the brass formation from the Cu diffractogram due to its reduced effect on the Cu lattice parameter. For this catalyst, the lattice parameters are already slightly increased at 220 °C (*a* = 3.634 Å) and 250 °C (*a* = 3.636 Å). The further increase to *a* = 3.647 Å at 400 °C is then again due to thermal expansion. However, due to the low intensity of the effect and concomitant thermal effect, it is difficult to draw a definite conclusion on possible alloy formation for the lowest Zn loading.

**Figure 4:**
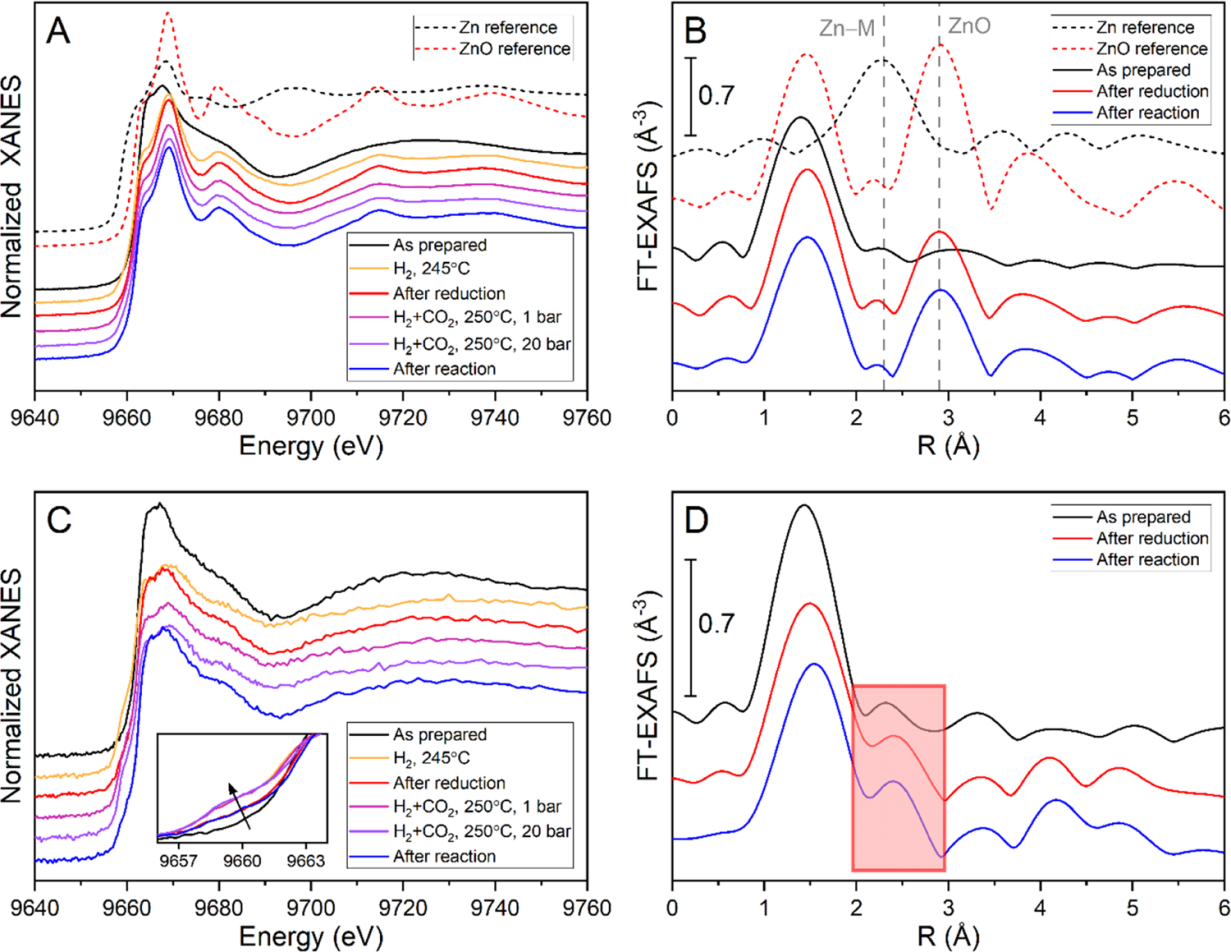
Zn K-edge (A, C) X-ray absorption near-edge structure (XANES) and (B, D) Fourier transformed extended X-ray absorption fine structure (EXAFS) of ZnO/Cu_2_O NC samples with (A, B) high and (C, D) low Zn loadings supported on SiO_2_. Reference lines in (B) are for the Zn–M (M = Cu, Zn) contribution for metallic Zn at 2.3 Å and for ZnO at 2.9 Å. The inset in (C) shows the pre-edge feature of the spectra without an offset. The red box in (D) highlights the evolution of a Zn–M contribution, indicating the formation of metallic Zn or a Cu–Zn alloy.

*Operando* XAS was then applied to get additional insight into the state of ZnO and its interaction with Cu. Since this technique is sensitive to short-range atomic ordering, it also detects noncrystalline phases. First, the samples were investigated in a packed bed flow reactor using a quartz capillary as the reactor tube. This allows for experiments under conditions very similar to those in the conventional reactor measurements, including high pressures up to 20 bar. To avoid the higher absorption caused by the ZrO_2_ support, the ZnO/Cu_2_O NCs were deposited on SiO_2_ instead of ZrO_2_. Furthermore, only the samples with the highest and the lowest Zn loading were measured. After measuring the initial state of the samples, an analogous reduction treatment in H_2_ as the one described above was applied at ca. 250 °C. Before changing to the reaction mixture, the sample was cooled down close to room temperature (<50 °C) to get high-quality spectra after the activation treatment without additional thermal disorder effects. Next, CO_2_ was introduced into the reactor and the sample was heated up to 250 °C in the reaction gas mixture (75% H_2_ + 25% CO_2_). First, some spectra were collected at atmospheric pressure, followed by data acquisition at 10 and 20 bar. After the reaction, high-resolution spectra were also recorded at room temperature. This series of spectra for each step are shown in Figure 4 for the Zn K-edge and in Figure S17 for the Cu K-edge. To ensure that the catalysts are working as they should, the outlet gas composition was measured with mass spectrometry. The results are shown in Figure S18. As expected, significant amounts of methanol (mass 31) can be detected only for the measurements at higher pressures (10 and 20 bar), guaranteeing the *operando* nature of the present investigation. As in the lab-based reactor measurements, the catalyst with the higher Zn loading showed a higher methanol yield as compared to the catalyst with the low Zn loading.

Analogous to our previous work with similar Cu_2_O NC catalysts without the ZnO overlayer,^40^ a reduction from Cu_2_O to metallic Cu is observed by XAS at the Cu K-edge during the H_2_ pretreatment (Figure S17). A significant contribution from Cu_2_O cannot be observed within the error margin of this method. This process occurs in a temperature range similar to that observed for the XRD measurements. During the whole experiment, the coordination numbers (CN) measured for Cu are close to those of bulk Cu, due to the large initial particle size of the Cu_2_O NCs (40–50 nm). The best-fit parameters for the Cu K-edge spectra are provided in Table S4. Fits of the EXAFS spectra for the catalysts with a high and low loading of Zn are shown in Figures S19 and S20, respectively. There are no significant differences in the Cu K-edge between the samples with different Zn loadings. Furthermore, no significant further changes were observed in the Cu K-edge spectra upon exposure of the catalyst to reaction conditions.

Because of the relatively large Cu particle size, the main signal of Cu is not coming from the surface of the particle, where the Cu is in close contact with the ZnO shell. Therefore, to obtain information about the Cu–Zn interaction and the general behavior of the Zn phase, one has to look at the Zn K-edge. Indeed, the evolution of the Zn K-edge spectra is different for both catalysts depending on the Zn loading. The XAS spectra showing this different behavior are displayed in Figure. 4. The initial XANES spectra of both samples are actually similar but do not represent either metallic Zn or ZnO in any of the most commonly known structures exactly. We assume that this is typical of the thin disordered layer, which was produced by our synthesis method on the Cu_2_O surface. A look at the initial EXAFS spectra shows only one peak at the position of the first shell Zn–O bonds. No peaks representing higher coordination shells could be clearly identified. This leads to the conclusion that initially ZnO is very disordered.

Once the samples are exposed to reducing conditions, the samples with the two different Zn loadings start to show distinct behavior. The XANES spectra of the high Zn loading catalyst closely resemble those of the bulk ZnO reference sample. Additionally, in the EXAFS spectra, an additional peak at 2.9 Å appears, corresponding to the first Zn–Zn bonds in ZnO. Also, there are indications of higher shell Zn–O bonds at >3.5 Å. This suggests that the initial ZnO structure on the surface of the Cu nanoparticles becomes more crystalline due to the reduction treatment in H_2_ (250 °C). The fits for all measurement conditions are shown in Figure S21, and the fit parameters are provided in Table S5.

In contrast, the Zn K-edge spectrum of the low Zn-loading catalyst does not change as drastically with respect to the as-prepared state. The shape of the XANES spectra still resembles that of the nanosized ZnO. As shown before, the XANES of nano-ZnO is distinct from the typical bulk ZnO.^32,44^ However, under the reducing conditions during the pretreatment and reaction, the XAS spectra of the low Zn loading catalyst become more similar to that of metallic Zn. In the XANES spectra, this is evidenced by a shoulder on the pre-edge that emerges (Figure 4C) upon the pretreatment in H_2_ already at 245 °C, and the general shape of the spectrum becoming flatter. Furthermore, in the EXAFS spectrum, a new feature shows up at around 2.4 Å, close to the position where the first shell Zn–Zn bonds are observed for metallic Zn or Zn–Cu bonds for a brass alloy. Because Cu and Zn are neighbors in the periodic table, it is hard to distinguish between these two possibilities, and therefore, Zn–Cu bonds and the formation of a Cu–Zn alloy (brass) cannot be excluded. Importantly, proper fitting of the EXAFS spectra can only be achieved by including this Zn–M (M = Cu, Zn) contribution. The corresponding fit curves for all conditions are shown in Figure S22. Nevertheless, there is still a very strong peak for the Zn–O bonds, indicating only an incomplete reduction, possibly to some form of partially reduced ZnO_*x*_ or ZnO coexisting with a Cu–Zn alloy. For the catalyst with the lowest Zn loading, no higher shell ZnO contributions appear, revealing that any ZnO species in this sample stay highly amorphous and also partially reduce during the activation treatment.

The changes that were introduced during the reduction in both samples also remain when changing to CO_2_ hydrogenation reaction conditions. We also were able to conclude a correlation between the state of Zn under reaction conditions, which is already established during the activation/reduction treatment, and the Zn loading on the catalyst. This means the oxidation state of Zn is driven not only by the reaction conditions but also by the initial amount of Zn. Furthermore, the pressure was changed from 1 to 20 bar under the reaction conditions. However, no significant influence of the pressure on the Zn or Cu K-edge spectra was found here.

In a follow-up experiment, a different experimental setup was used, which allowed the measurement of the ZnO/Cu_2_O NCs on the ZrO_2_ support. This control data set served to ensure that the results obtained in the previous experiment for the samples supported on SiO_2_ are transferable for the NCs supported on ZrO_2_. During these measurements also, the catalyst with a medium Zn loading was measured. This sample, however, did not change significantly after exposing it to reaction conditions, but remained as disordered ZnO. These additional measurements and results are described in detail in Supplementary Note 5.

Summarizing these results, one can conclude that having less ZnO on the Cu surface leads to a higher degree of reduction of ZnO. This leads to the formation of a Cu–Zn alloy at the Cu–Zn interface. At higher initial amounts of ZnO in the precatalyst, no clear additional reduction is observed in the XAS spectra with respect to the initial as-prepared state. However, we cannot completely exclude the formation of an alloy, but this might no longer be observed due to the higher proportion of ZnO. At the same time, the high loading catalyst shows the formation of a ZnO overlayer with a more ordered crystalline structure instead of the initial disordered one during the reaction.

## Discussion

**Figure 5:**
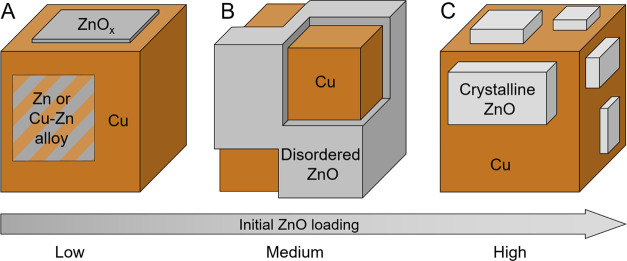
Schematic model of the final working state of the ZnO/Cu_2_O NC precatalysts during CO_2_ hydrogenation. The behavior is different for (A) low, (B) intermediate, and (C) high loadings of Zn, which significantly influences the final Zn oxidation state and structure.

In the following, we link the results of the structural and chemical analyses to the reactivity of the catalysts. Initially, the catalysts differ only in the amount of Zn that is used during their synthesis. From the results of our measurements, we can however see that the catalysts, which only differ by the initial Zn loading, show a quite different behavior in terms of the structure and oxidation state of Zn, as well as the Zn distribution on the surface of the initial Cu_2_O nanocubes and later on NPs. Other than the distinct morphological structures unveiled by the TEM–EDX images, additional changes of the chemical state become evident under reducing conditions during the pretreatment. For the low Zn loading samples, ZnO partially reduces, while for the high Zn loading samples, Zn preferentially stays oxidized and becomes more crystalline. The ZnO reduction for the low Zn-content samples was associated with the formation of Cu–Zn alloys. This should have direct consequences for the catalytic performance of these catalysts, and indeed, the catalytic activity clearly differs depending on the chosen loading. A model is presented in Figure 5 showing the structure of the catalysts with different Zn loadings.

The low loading catalysts show basically no improvement in activity compared to the pure pre-reduced Cu_2_O NCs. Therefore, the reduced ZnO species, or rather a Cu–Zn alloy is not beneficial for the methanol synthesis. On the other hand, when sufficient ZnO is available on the Cu_2_O NCs and no drastic ZnO reduction takes place, as is the case for the intermediate Zn loading, an increase in activity is obtained (Figure 2). Following this trend, one would expect that the catalyst with the highest Zn loading should also have the highest activity. Knowing that having Cu and ZnO in close contact is good for the catalytic activity, one might assume that more Zn would result in more active sites.^27^ This is actually not the case, but instead, no improvement compared with the medium loading is observed. This means that there is an optimal loading of Zn on the catalyst. Indeed, there are studies showing the dependence of the activity on the Zn content in Cu/ZnO catalyst^5^ or the Zn coverage on Cu surfaces^26,45,46^ and that there is an optimal ratio, where the optimal amount of Zn is given at a Zn coverage of about 20% of the Cu surface.^26,45,46^ In addition, multiple studies report that a high degree of crystallization, especially of ZnO, is detrimental to the catalytic activity of the Cu/ZnO system.^47−49^ It should be noted that these reports have a gas feed containing CO, although being CO_2_-rich. We also observe higher crystallization for high loading and the formation of larger ZnO particles. Thus, although more ZnO is present, not all of it would be in direct contact with Cu and would profit from the Cu–ZnO synergy.

Regarding the oxidation state, it has been reported that under certain reaction conditions, Cu–Zn alloys could form,^32,40^ but also that they might be unstable and might transform into ZnO and Cu under CO_2_ hydrogenation conditions.^14,23^ To create an alloy often higher temperatures and stronger reducing atmospheres are needed so that even if brass is formed during the activation pretreatment, it might not be stable in a H_2_ and CO_2_ mixture around 250 °C and it is certainly not needed to explain the high activity of the Cu–ZnO interface.^13,50^ Our XRD data also show that Cu–Zn alloys can be formed and stabilized at high reaction temperatures (e.g., at 400 °C in H_2_ + CO_2_). Thus, the oxidation state of Zn can be tuned by multiple factors, such as the initial Zn loading, reaction pretreatment, temperature, and pressure.^32,40,51^ In addition, it should be noted that the addition of CO to the feed gas mixture, which is typical in industrial methanol synthesis, may also stabilize reduced Zn species.^7,52^ Considering all of these different factors, one should note that the reaction conditions can be used to tune the state of the catalysts and thus should be considered when pushing the catalyst into the desired state under reaction conditions. This is important to understand because the interplay of the reaction conditions, the chemical state, and structure of the catalyst may be able to unlock different reaction pathways.^52,53^ However, the actual state of the catalyst during the reaction will always be influenced by both the reaction conditions and the initial precatalyst structure and composition. In fact, the present study reveals that under the right conditions, some reduced Zn may actually be stable in H_2_ + CO_2_ because the oxidation state of Zn and ease of Cu–Zn alloy formation appear to correlate with the Zn coverage on the Cu surface, which we have carefully tuned here through our chemical synthesis of ZnO overlayers on the surface of Cu_2_O NCs. Similar behavior was also reported before for other material systems, where the Zn oxidation state after the reaction was found to change depending on the Zn coverage on the Cu surface.^20,22,53^ In one case, the change to the more oxidized Zn state was less active, but also heavily dependent on the Cu surface structure.^26^ Furthermore, regardless of whether the different studies claim Cu/ZnO^23^ or a Cu–Zn alloy^22^ as the active phase, there seems to be an optimal Zn coverage of about θ_Zn_ = 0.2. Stabilizing this intermediate state is critical for achieving high catalytic activity. The presence of reduced Zn cannot be confirmed in our high Zn loading catalysts. However, this does not exclude the possibility that small amounts of reduced Zn are still there. Instead, the low contribution of a possible Cu–Zn alloy is hidden under the high amount of ZnO. Nevertheless, it is clear that the fraction of ZnO is definitely higher, as is its crystallinity and bulk-like ZnO structure during reaction.

Thus, our data clearly demonstrate that brass formation is detrimental to the methanol synthesis performance and that ZnO is needed for an active Cu/ZnO catalyst, but one should also optimize their interfacial contact area, which was achieved here by employing cubic Cu_2_O particles overcoated with thin ZnO layers as precatalysts. This approach mimics the strong metal–support interaction effect that takes place during reduction and under reaction conditions for the industrial catalysts,^9,54^ where it effectively leads to a distorted ZnO film, graphitic-like ZnO or less strongly oxidized Zn^δ+^ species^15^ on the surface of the Cu NPs. In the traditional Cu NP/ZnO catalysts, where bulk ZnO is available as support, the progressive migration of ZnO onto the Cu NP surface during extended reaction times might lead to thicker ZnO films and to their recrystallization, and thus, to partial deactivation of the catalysts.^30^ This is in fact what we see here when we compare the medium and high Zn loadings on the Cu_2_O NPs where we have highly disordered versus crystalline ZnO overlayers during the reaction, respectively. Furthermore, if the choice of the initial precatalyst or if the reaction conditions allow the stabilization of Zn–Cu alloy species, a decrease in the activity would take place.^32^ Nonetheless, it should be noted that although metallic Zn in Cu–Zn brass alloys is not as effective in promoting the methanol synthesis as Cu/ZnO, it is still better than bare Cu.^32^

The actual nature of the active species for this reaction is, in fact, very hard to unveil, as ZnO appears to dynamically adapt its structure to the given reaction conditions. Even the disordered ZnO may potentially only be a precursor that will adapt its structure once it comes into contact with the reactants or intermediates. However, it seems that a high amount of disordered ZnO in close contact with metallic Cu leads to high activity, even if it might dynamically change between a Cu–Zn alloy, Zn-formate, or ZnO temporarily.^23,55^

Additionally, it should be noted that for our reactivity studies, the Zn-covered Cu particles were mixed with ZrO_2_ to suppress sintering of the Cu particles. There might be an interaction of Cu and Zn with the ZrO_2_, which can also influence the catalytic activity.^56^ An indication of a Cu–ZrO_2_ interaction leading to methanol formation is the similar activity of the ZrO_2_-supported but Zn-free Cu_2_O NC catalyst and the low Zn-loaded Cu_2_O NC precatalyst, despite the Zn-free catalyst usually being less active than Zn-containing ones even if the Zn loading is low.^53^

In general, the oxidation state and structure of Zn are heavily dependent on multiple factors such as the reaction gas mixture, pressure, temperature, time under operation, Zn surface coverage, or degree of ZnO crystallization. These factors can critically influence the reactivity and methanol yield. Moreover, as mentioned above, the dynamic nature of the ZnO/Cu reactive interface further complicates its understanding, since most of the available studies are based on information on the material before/after reaction or under nonrealistic reaction conditions or were performed without sufficient temporal resolution. Therefore, it is hard to make a meaningful comparison of the extensive data available in the literature on this reaction and material system unless they were obtained under comparable experimental conditions. In particular, some analysis methods might not be applicable under the high pressures required to obtain significant methanol yields, leading to results that might be influenced by the pressure gap. Furthermore, it is also necessary to take into account that catalysts evolve under reaction conditions, which is why *in situ* and *operando* characterization methods are mandatory to gain insight into strongly evolving systems such as Cu/ZnO. Identifying the correct surface structure under reaction conditions, as it was attempted here, also provides fundamental information for an accurate theoretical modeling of this system, which certainly cannot be exclusively based on the structure and surface composition of the experimental precatalyst material.

## Conclusions

In summary, cubic Cu_2_O nanoparticle precatalysts with a ZnO shell of adjustable thickness were synthesized, which allowed starting from a point where Cu and Zn are already in close contact with each other. The combination of microscopic and spectroscopic methods with reactivity measurements allowed us to link the catalytic performance to the structure of the catalyst under reaction conditions. More specifically, the initially disordered ZnO becomes more heavily reduced, forming a Cu–Zn alloy at lower ZnO loadings and more crystalline ZnO at high Zn loadings. These changes have profound consequences for the catalytic performance. The catalysts with a low Zn loading and Cu–Zn alloy species present under reaction conditions do not show any improvement in the catalytic performance as compared with Zn-free Cu particles on ZrO_2_. This indicates that a certain minimum amount of ZnO has to be present in the precatalyst so that subsequently under reaction conditions ZnO species (ideally highly disordered), are still available in close contact with Cu. Under the present experimental conditions, the formation of brass observed for the low initial Zn loadings does not lead to an enhanced catalytic activity. Moreover, high initial Zn loadings characterized by a strongly crystallized ZnO and ZnO agglomerates on Cu, also result in an inferior methanol yield as compared to the medium Zn loadings. This is assigned to the partial loss of the interfacial contact area and the possible detrimental effect of the bulk-like ZnO overlayer. Thus, our study reveals that an optimum initial Zn content, leading to a homogeneous coverage of highly disordered ZnO on Cu (medium Zn loading here), is required to achieve high methanol yields in CO_2_ hydrogenation.

It is necessary to note that by modifying one of the parameters of this material system, for example, the initial precatalyst particle size or shape, or the location, content, and distribution of the Zn species, one will also affect the formation and reactivity of the highly dynamic Cu–ZnO interface. Here, we kept the initial Cu particle size and shape (cubic) identical and also attempted to stabilize the Zn species as ZnO through the initial interface with Cu_2_O, as well as by tuning in the synthesis the Zn coverage on the Cu surface of the precatalyst. Our experimental results highlight that not only the reaction conditions but also the amount of initially available Zn plays a crucial role in the formation of the active Cu–Zn interface under working reaction conditions. In particular, starting Zn loadings leading to thin disordered but stable and homogeneous ZnO coverages on Cu during the reaction are preferred. Transferring these results to an industrial-type catalyst, prepared by coprecipitation of Cu and Zn, would mean that too high Zn loadings in such catalysts must be avoided since they can lead to the presence of highly crystalline ZnO domains onto the Cu surface with a decreased reactivity.

## Supporting Information Available

Additional remarks for the background in the XRD measurements; note on additional XAS measurements; additional remarks on the XPS measurements together with fitted XPS spectra; calculations for the estimation of the ZnO shell thickness; note on ZrO_2_ support and XRD pattern of the used ZrO_2_ support (Notes 1–5); exemplary XANES spectrum of Zn K-edge and the Hf L_3_-edge for a ZnO/Cu/ZrO_2_ catalyst; additional STEM data (images, size histogram, EDX maps, and spectra); selectivity of the catalysts; *in situ* XRD spectra of catalysts during reduction; mass spectrometry data from *operando* XAS measurements; additional XAS data (XANES and FT-EXAFS spectra, fitted FT-EXAFS curves) (Figures S1–S24); and fitting parameters extracted from XRD Rietveld and XAS data analysis (Tables S1–S5) (PDF)
