# Supplementary material for: Enhanced Methanol Synthesis from CO2 Hydrogenation Achieved by Tuning the Cu–ZnO Interaction in ZnO/Cu2O Nanocube Catalysts Supported on ZrO2 and SiO2
Source: J Am Chem Soc. 2024 Mar 12;146(12):8677–87. doi: 10.1021/jacs.4c01077 (PMC10979448; doi:10.1021/jacs.4c01077)
Supplement: Supplementary file 1 — ja4c01077_si_001.pdf [file ja4c01077_si_001.pdf]

Supporting Information for

Enhanced Methanol Synthesis from CO_2 hydrogenation achieved by tuning the Cu-ZnO interaction in ZnO/Cu_2O Nanocube Catalysts supported on ZrO_2 and SiO_2

*David Kordus[1,2], Simon Widrinna[1,2], Janis Timoshenko[2], Mauricio Lopez Luna[2], Clara Rettenmaier[2], See Wee Chee[2], Eduardo Ortega[2], Osman Karslioglu[2], Stefanie Kühl[2] and Beatriz Roldan Cuenya[2]**

[1] Department of Physics, Ruhr-University Bochum, 44780 Bochum, Germany

[2] Department of Interface Science, Fritz-Haber Institute of the Max Planck Society, 14195 Berlin, Germany

* Corresponding author: roldan@fhi-berlin.mpg.de

Supplementary Note 1: Additional remarks for XRD measurements

Additional peaks are seen in the XRD patterns that originate from the background of the reaction cell used for these measurements. To identify these peaks, reference measurements were performed using a pure ZnO powder and a metallic Cu foil. The reference measurements were done at room temperature in the reaction cell filled with He. A comparison of the two reference samples with one of the catalysts (ZnO/Cu_2O NCs with high Zn loading, after reaction) is shown in Fig. S1.

One can see that there are multiple peaks in the XRD pattern from the references overlaying with each other. Those are the peaks originating from the background of the sample holder, since metallic Cu and ZnO should have no peaks at the same positions. The features at 31.8° and 36.2° do correspond to the (100) and (101) reflections of ZnO and are found at position where there is no contribution from the background seen in the Cu reference pattern. Although there is one peak from the background at ~35.9° close to the ZnO peak at 36.2°, these peaks can be identified as two distinct components. Importantly, the peak at ~34.1° mostly originates from the background and not from ZnO, although it does overlap with the ZnO(002) reflection and probably includes some contribution of this.

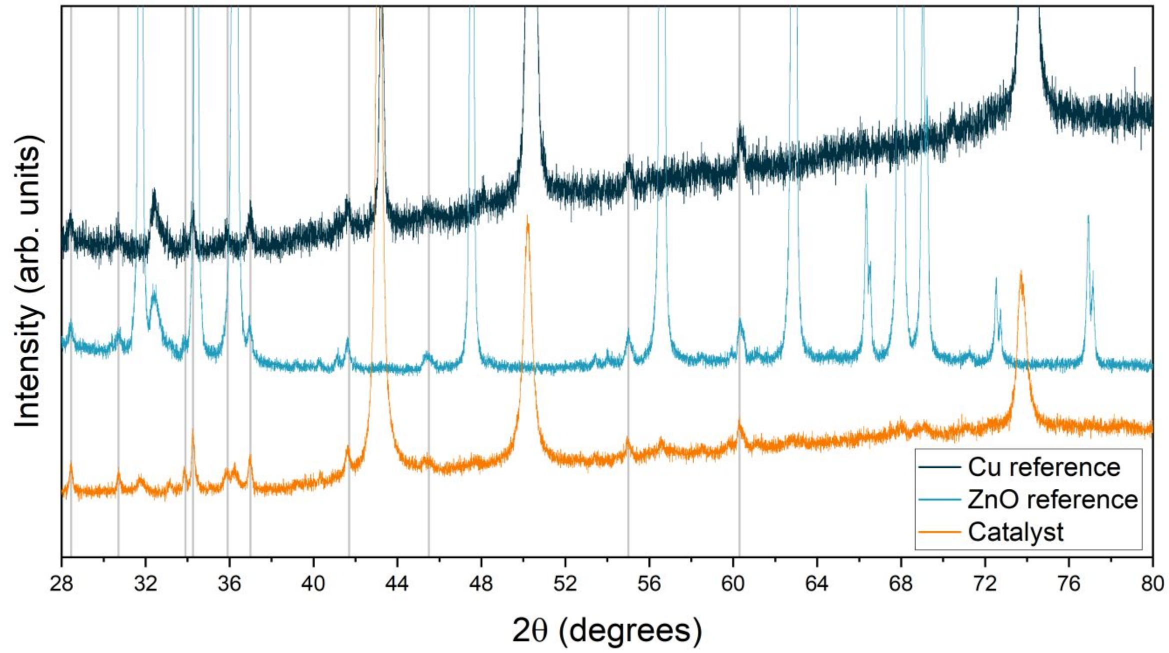

Fig. S1 Comparison of the XRD patterns from a metallic Cu reference, a ZnO reference and the catalyst with high ZnO loading after reaction. Peaks appearing for all three samples that are indicated by the vertical grey lines stem from the background of the sample holder.

Supplementary Note 2: Additional XAS measurements

The XAS results presented in the main text were obtained in a flow reactor configuration using a quartz capillary. During these measurements the Cu_2O/ZnO NC catalysts were supported on SiO_2. Therefore, follow up experiments were done to ensure that the results obtained in the previous experiment for the samples supported on SiO_2 are transferable to the NCs supported on ZrO_2. Thus, particles supported on both materials (SiO_2 and ZrO_2) were measured under the same conditions and compared with each other. These additional XAS measurements were performed at the CLÆSS beamline of the ALBA synchrotron in a solid-gas reactor cell[1] (ITQ) provided by the beamline. Here, the samples were diluted with boron nitride for optimal signal and pressed into pellets. Samples were measured in transmission or fluorescence modes, depending on the Zn loading on the respective samples. Specifically, the samples with low Zn loadings had to be measured in fluorescence, because the high amount of Cu and the support were too absorbing to achieve good transmission signals.

When measuring the Zn K-edge (9659 eV) on the ZrO_2 supported samples, the spectra were overlaid with an additional signal coming from the Hf L_3-edge (9561 eV), which is known to be a common contaminant in ZrO_2. The Hf edge is relatively small and far enough away from the Zn edge, so it does not influence the quality of the obtained spectra in a meaningful way. An example of the Zn and Hf edges together is shown in Fig. S2.

Because the previous results showed that the catalysts did not significantly change when they were exposed to reaction condition after the activation treatment in H_2 was completed, the catalysts were only exposed to H_2 at ca. 250°C and then cooled down to room temperature. The spectra of the Cu and Zn K-edges, that were recorded before, during and after the reduction, are shown in Figs. S23 and S24.

The spectra obtained during the reduction look the same as the spectra afterwards, just with the added disorder due to the higher temperature. Analogously to the SiO_2-supported NCs, the XANES and EXAFS spectra of the Cu K-edge of the ZrO_2-supported NPs show a reduction from Cu_2O in the initial state to metallic Cu after the reduction treatment. Because of the relatively large size of the Cu particles (approx. 40 nm) the Cu edge looks very similar to the Cu foil reference (bulk, metallic Cu). Importantly, no differences between the particles supported on SiO_2 and ZrO_2 can be detected for the Cu K-edge. Therefore, at least from the point of view of the Cu edge, these catalysts behave the same. Also, no influence from the different Zn loadings on the reduction of the Cu particles can be observed in the Cu K-edge spectra.

Regarding the Zn K-edge, the samples with the high and low Zn loadings exhibit similar behavior as in the previous experiments on SiO_2 (Fig. 4 in main text). The effects are a bit less pronounced this time, presumably caused by different reaction cell geometries and the dilution with BN. For the high loading sample after reduction, the XANES spectra look more like the bulk ZnO reference and also the EXAFS spectra show the appearance of peaks corresponding to higher coordination shells of ZnO, indicating a more crystalline structure. The spectra from the low loading samples indicate a partial reduction of ZnO instead. This can be concluded from the shape of the XANES spectra as well as the appearance of additional

peaks in the EXAFS spectra at a position, typical for the Zn-Zn or Zn-Cu bonds of metallic Zn or a Cu-Zn brass alloy, respectively.

Furthermore, the experiments were conducted on samples with all three of the different Zn loadings that were used for reactivity measurements, including the catalyst with the medium Zn loading. Interestingly, for the intermediate Zn loading, also the Zn K-edge XANES spectra do not change much during the activation treatment. Additionally, no real indication for reduction, but also no contribution from higher coordination shells are observed in the Zn K-edge EXAFS spectra. This implies that this catalyst mostly can preserve its initial structure even after the reduction treatment, meaning some form of disordered ZnO with low crystallinity. Finally, also for the Zn edge no major differences were observed between the two support materials for all catalysts.

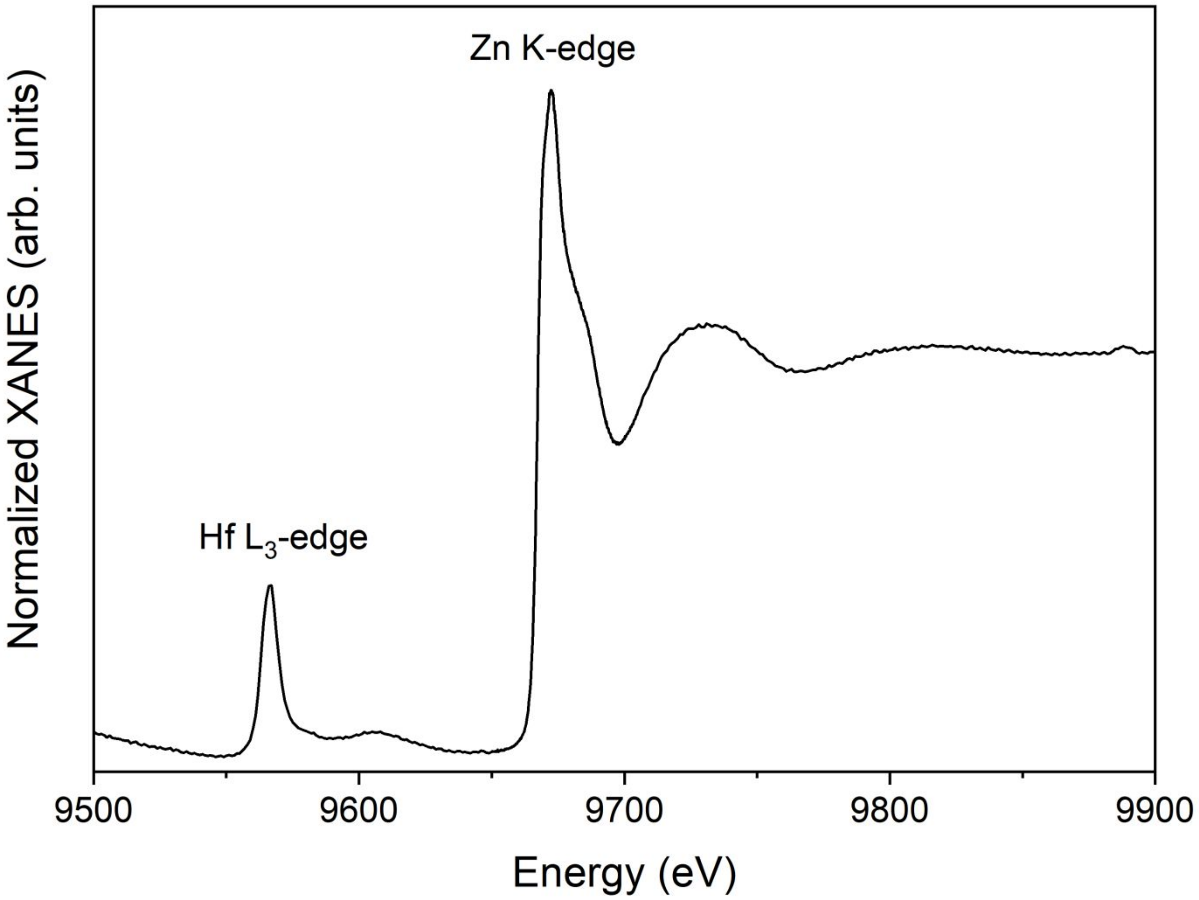

Fig. S2 XANES spectrum of the Zn K-edge and the Hf L_3-edge for the core-shell Cu_2O/ ZnO catalyst deposited on ZrO_2 catalyst with the lowest Zn loading. Hf is a common contaminant in the ZrO_2 support material, which makes about 70% of the whole sample.

Supplementary Note 3: Additional remarks for XPS measurements

XPS spectra of the Cu 2p and Zn 2p regions for the Cu_2O NCs with different ZnO loadings are shown in Fig. S3. The increasing amount of ZnO in the samples is represented by an increasing area of the ZnO peaks. Relatively high amounts of Zn compared to Cu are observed due to the surface-sensitive nature of XPS and that ZnO is located at the surface of the Cu_2O nanocubes.

Additionally, Cu is not only present as Cu_2O, but also as CuO, indicated by the large satellite at 938 – 947 eV. Because the samples are transferred in air to the XPS system, an oxidation from Cu_2O to CuO at the surface of the NCs is expected. Interestingly, the ratio of the two different Cu oxides is also dependent on the ZnO loading. The composition of the samples is shown in Table S1. It seems that the oxidation state of the Cu_2O NCs is better preserved underneath a thicker, protective ZnO layer.

**Zn content**	**Cu_2O (at%)**	**CuO (at%)**	**ZnO (at%)**	**Cu_2O/CuO ratio**	**Zn/Cu ratio**
No Zn	29.7	70.3	0	0.42	0
Low	27.6	68.6	3.8	0.40	0.04
Medium	49.1	29.8	21.1	1.65	0.27
High	29.2	17.2	53.6	1.70	1.16

Table S1 Relative composition of the different Cu and Zn species in as-prepared ZnO/Cu_2O NC catalysts with different ZnO loadings.

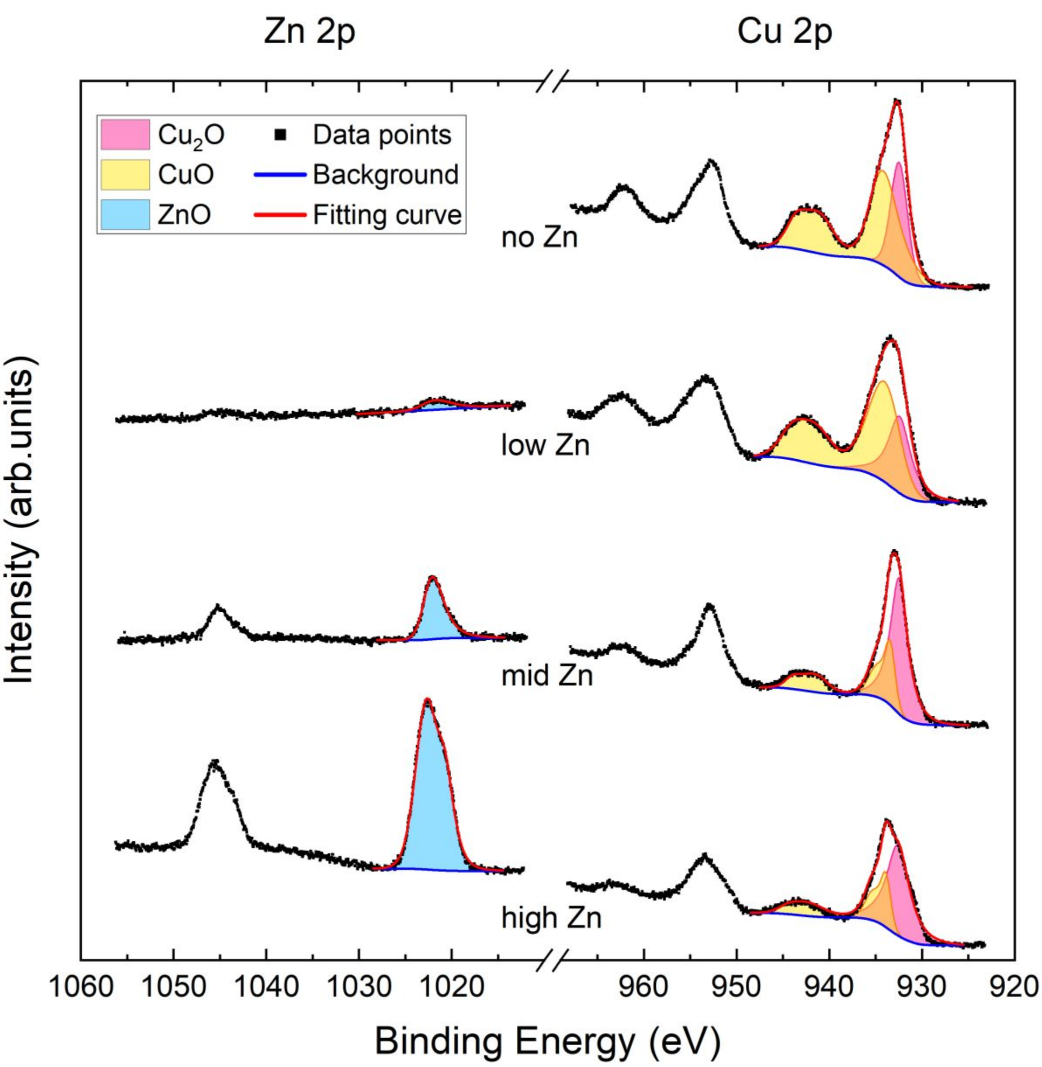

Fig. S3 Cu 2p and Zn 2p XPS spectra for the as prepared ZnO/Cu_2O NCs with no Zn, low, medium and high loadings of Zn. The nanocubes in these catalysts are supported on ZrO_2.

Supplementary Note 4: Estimation of the ZnO shell thickness

To estimate of the thickness of the ZnO shell from the ICP-MS experiments we will assume that we have a perfect Cu_2O cube with edge length *l* that has a homogenous layer of ZnO with thickness *t*. Furthermore we assume that the thickness of the overlayer *t* is small compared to the edge length *l* of the cube. The volume occupied by the ZnO shell is then given by $V_{shell} = (l + 2t)^3 - l^3$. Furthermore, we know $V_{shell} = m_{\mathrm{ZnO}}\, m_{\mathrm{Cu_2O}}^{-1}\, \rho_{\mathrm{Cu_2O}}\, \rho_{\mathrm{ZnO}}^{-1}\, l^3$, where ρ is the density of the material. This leads to the formula:

$$t = \frac{1}{2} l \left(\sqrt[3]{1 + \frac{m_{\mathrm{ZnO}}\, \rho_{\mathrm{Cu_2O}}}{m_{\mathrm{Cu_2O}}\, \rho_{\mathrm{ZnO}}}} - 1 \right)$$

The edge length *l* is given average edge length measured with STEM (46 nm). The ratio $m_{\mathrm{Zn}}/m_{\mathrm{Cu}}$ is given by the ICP measurements (Tab. 1 in the main text), so one can calculate the corresponding$m_{\mathrm{ZnO}}/m_{\mathrm{Cu_2O}}$ratios. The resulting shell thicknesses are: $t_{\mathrm{lowZn}} = 0.1\ \mathrm{nm}$, $t_{\mathrm{mediumZn}} = 0.6\ \mathrm{nm}$ and $t_{\mathrm{highZn}} = 2.6\ \mathrm{nm}$.

As an alternative, we can also estimate the ZnO overlayer thickness *t* from the experimental XPS results. Cumpson[2] provided a numerical way to calculate the layer thickness *t* from XPS measurements based on the formula:

$$\ln\left(\frac{I_o/s_o}{I_s/s_o}\right) - \left[\left(\frac{E_o}{E_s}\right)^{0.75} - \frac{1}{2}\right]\frac{t}{\lambda_o \cos\theta} - \ln 2 = \mathrm{lnsinh}\left(\frac{t}{2\lambda_o \cos\theta}\right)$$

Here, I_o and I_s are the measured peak intensities from the overlayer and substrate, respectively, together with the corresponding sensitivity factors s_o and s_s. Therefore, the factor $\left(\frac{I_o/s_o}{I_s/s_o}\right)$ is basically just the Zn/Cu ratio given in table S2. E_o and E_s are the peak kinetic energies. λ_o is the attenuation lengths of photoelectrons within the overlayer which can be calculated with the TPP2M formula[3] to λ_o = 11.62 Å. The emission angle θ is normal to the surface here and therefore, $\theta = 1 (\cos\theta = 1)$. Solving the equation numerically then gives the following results for the shell thicknesses: $t_{\mathrm{lowZn}} = 0.05\ \mathrm{nm}$, $t_{\mathrm{midZn}} = 0.28\ \mathrm{nm}$ and $t_{\mathrm{highZn}} = 0.96\ \mathrm{nm}$.

From this we see that the values obtained for the ZnO layer thickness from the XPS data are consistently lower than those calculated with the ICP results. This can be attributed to the formation of a layer with inhomogeneous thickness. In both models actually a homogenous ZnO layer on the surface of the Cu_2O cubes is assumed. But, because XPS is surface sensitive, an inhomogeneous layer with regions of thicker ZnO and others with partially exposed Cu_2O/Cu will lead to an overall lower ZnO layer thickness. That this might be the case can also be observed from our STEM measurements (Fig. 1B in the main text).

Supplementary Note 5: Choice of ZrO_2 as a support material

The industrial methanol synthesis process uses a $Cu/ZnO/Al_2O_3$ catalyst, where Cu and ZnO form the active site.[4–6] The gas feed is composed of $H_2/CO/CO_2$ and even though CO is used in the feed gas, CO_2 was identified to be the main carbon source for methanol synthesis.[7–9] One purpose of using CO is to remove water, which is produced simultaneously to methanol and it is suggested to block the surface of the catalyst.[10] For the direct conversion of CO_2 to methanol in a CO-free feed, ZrO_2 containing catalysts are frequently used.[11] ZrO_2 was found to be a good support for Cu[12,13] due in part to the weak hydrophilicity of ZrO_2.[14] Additionally, the use of ZrO_2 is thought to result in an increase of the activity and/or selectivity by increasing the Cu dispersion[15–17], stabilizing certain Cu nanoparticles (NPs),[11,18,19] by modifying surface properties such as acidity[20], or by influencing the adsorption of H_2, CO_2[21] and reaction intermediates[22]. It was also observed that the crystal system (amorphous, monoclinic or tetragonal) of ZrO_2 has also an influence on the activity.[20,23–25] An important factor for a good catalyst is often a good contact of Cu and ZrO_2 with a high interfacial area.[26–28] For ternary $Cu/ZnO/ZrO_2$ catalysts,[29,30] the system becomes even more complex compared to the Cu/ZrO_2 system due to the additional ZnO-ZrO_2 and Cu-ZnO interactions.

The ZnO/Cu_2O NCs were supported on a ZrO_2 to reduce the NC sintering. XRD measurement of the support material (Fig. S4) unveiled that the ZrO_2 used is mostly in its more stable monoclinic form, with only minor contributions from other crystalline ZrO_2 phases: 99.5% monoclinic and 0.5% tetragonal/cubic ZrO_2. It is important to highlight here that the main monoclinic phase is not expected to create additional acidic centers[20] (as reported for tetragonal ZrO_2). Thus, the ZrO_2 support should not influence the catalytic performance of the ZnO/Cu_2O NCs in a major way.

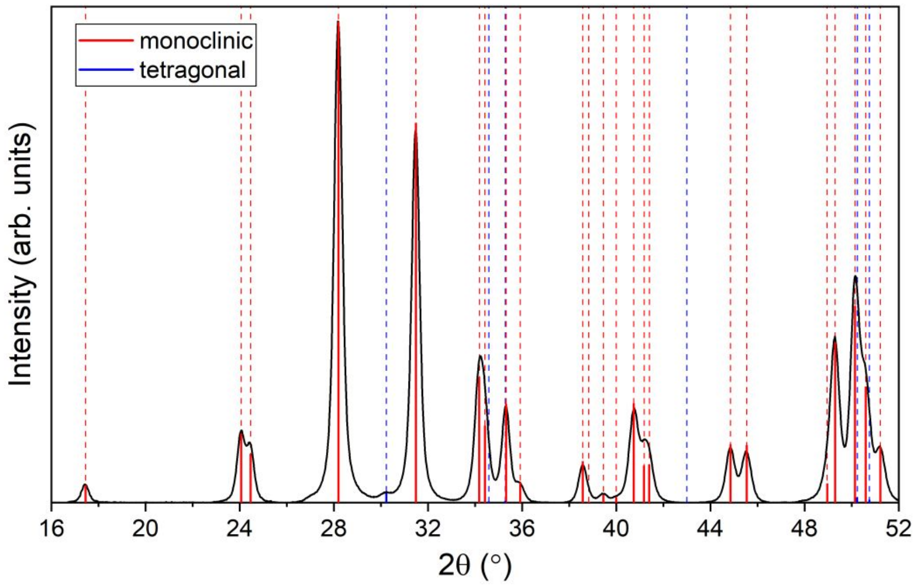

Fig. S4 XRD pattern of the ZrO_2 used as a support for the Cu_2O/ZnO nanocubes together with peak reference lines for monoclinic (red) and tetragonal (blue) ZrO_2.

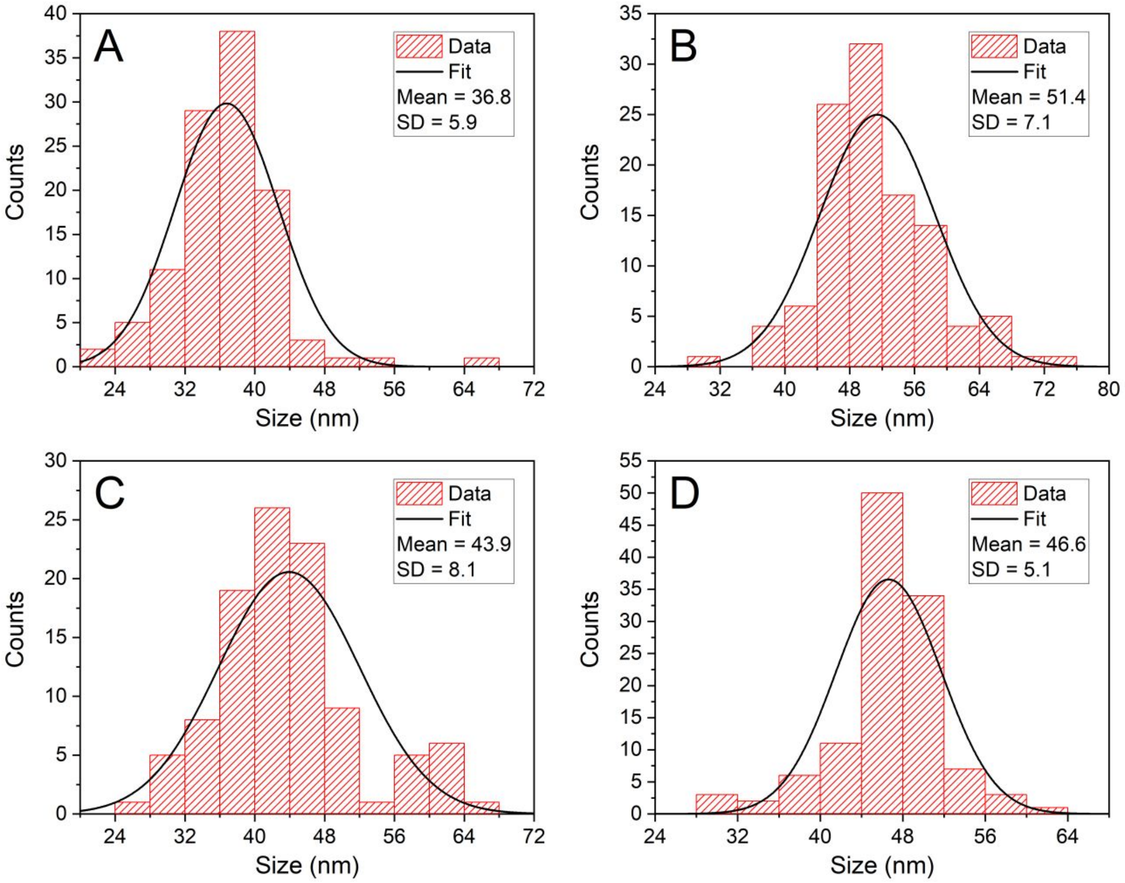

Fig. S5 Particle size histograms for Cu_2O nanocube particles with and without a ZnO shell. The histograms were created from multiple images and samples. Size refers to the edge length of the particles. The average particle size is (A) 37 ± 6 nm for the Cubes without Zn, (B) 51 ± 7 nm for Cubes with low Zn loading, (C) 44 ± 8 nm for Cubes with medium Zn loading and (D) 47 ± 5 nm for Cubes with high Zn loading.

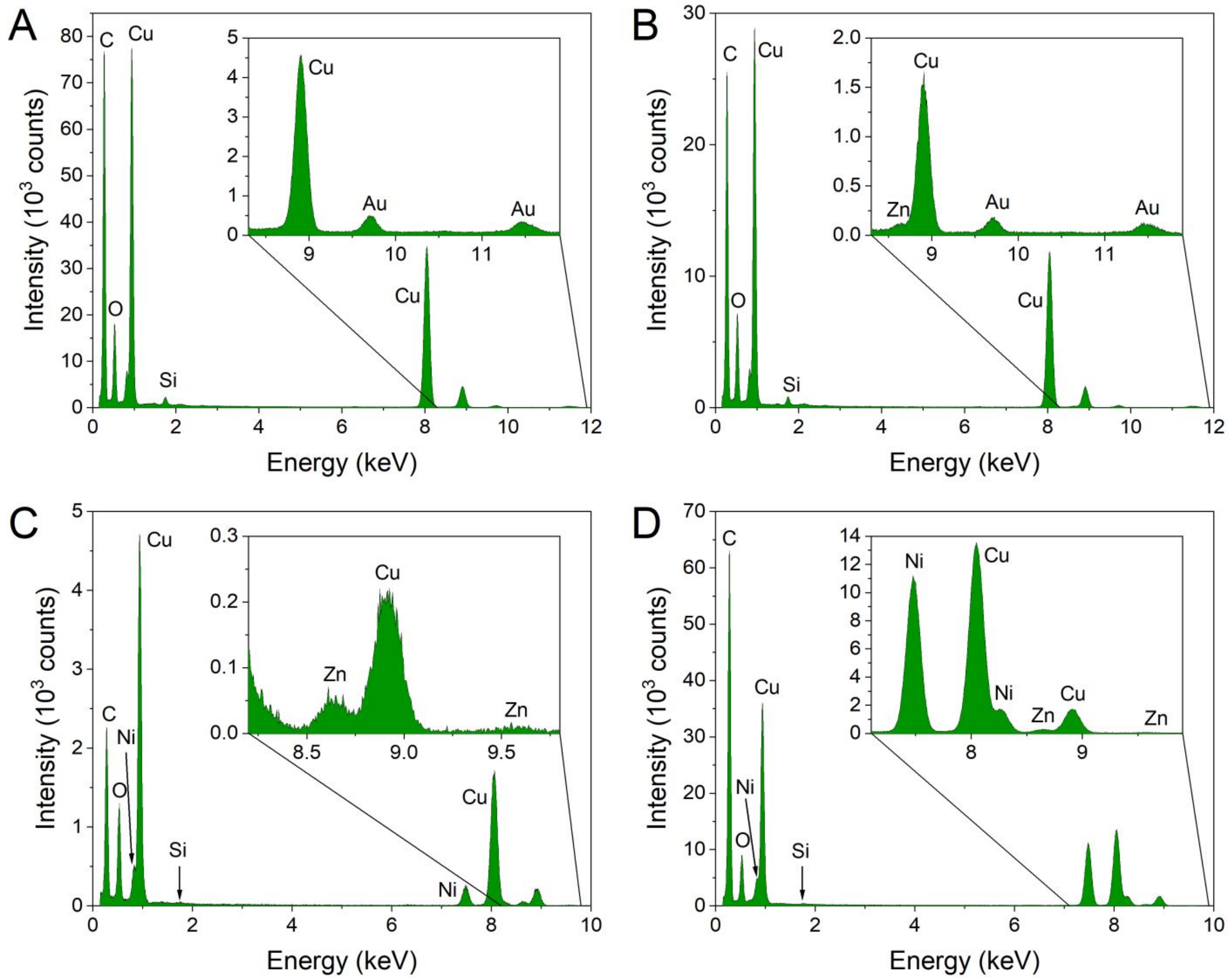

Fig. S6 EDX spectra for the corresponding EDX maps in figure 1 of Cu_2O cubes with (A) no Zn, (B) low, (C) medium and (D) high amounts of Zn. For the measurements, samples were supported on lacey-carbon gold or nickel grids and therefore, additional peaks for Ni (A, B) and Au (C, D) appear.

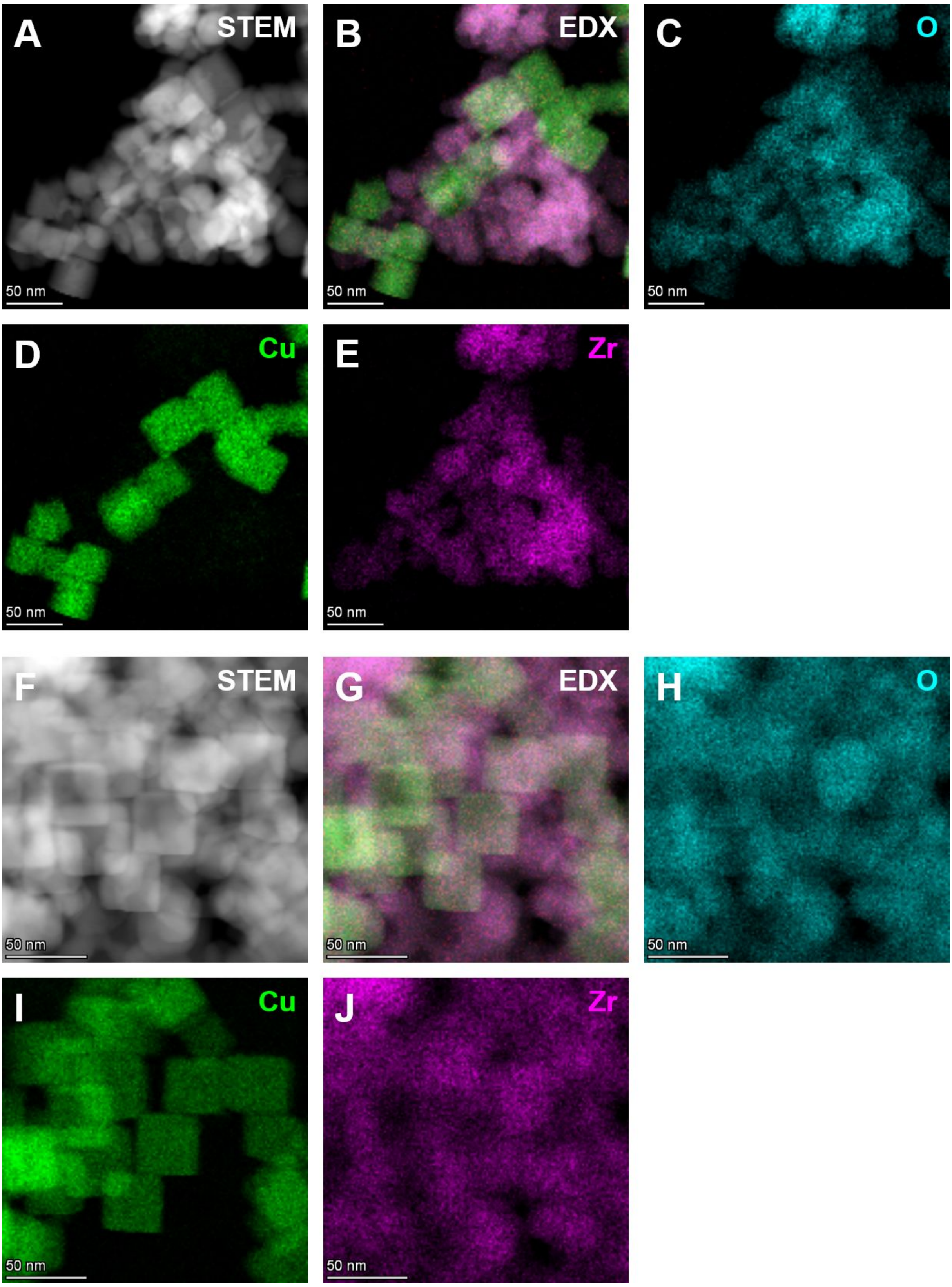

Fig. S7 STEM images and EDX maps of the as prepared Cu_2O NCs supported on ZrO_2. Shown are (A, F) the STEM dark field image, (B, G) combined EDX maps of multiple elements (Cu in green and Zr in magenta) and (C-E, H-J) the individual EDX maps of the indicated elements.

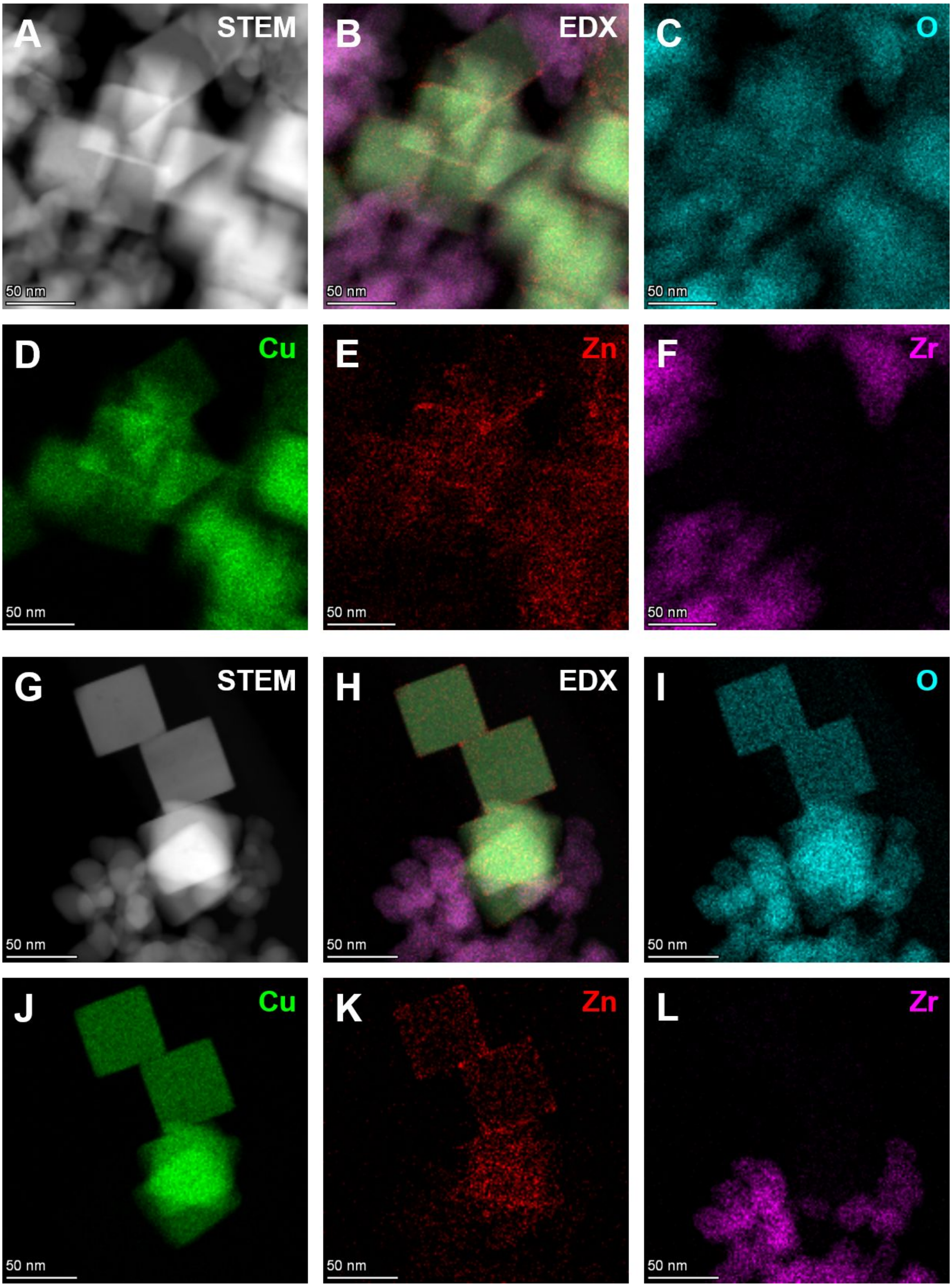

Fig. S8 STEM images and EDX maps of the as prepared ZnO/Cu_2O NCs with the low Zn loading supported on ZrO_2. Shown are (A, G) the STEM dark field image, (B, H) a combined EDX maps of multiple elements (Cu in green, Zn in red and Zr in magenta) and (C-F, I-L) the individual EDX maps of the indicated elements.

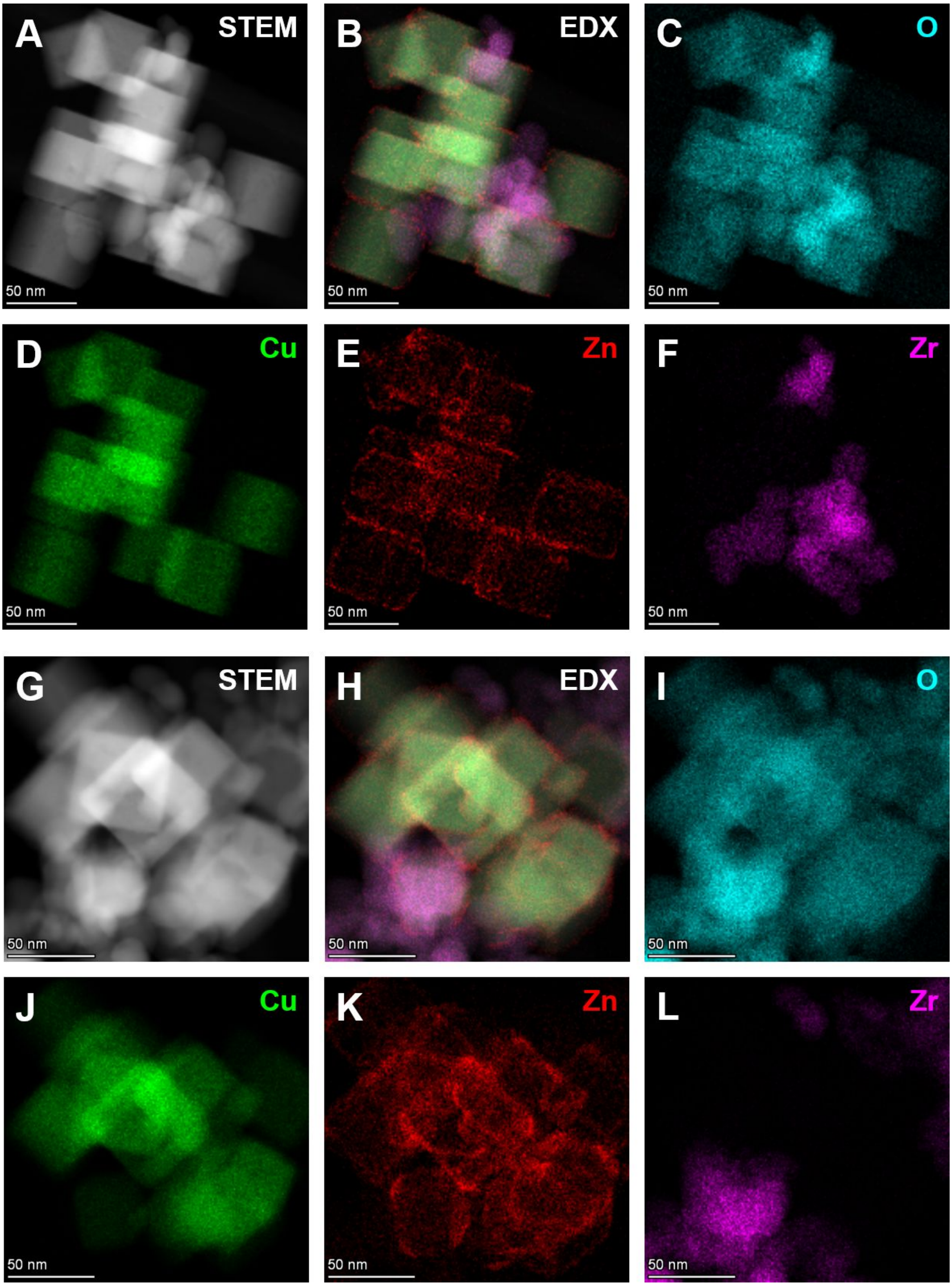

Fig. S9 STEM images and EDX maps of the as prepared ZnO/Cu_2O NCs with the medium Zn loading supported on ZrO_2. Shown are (A, G) the STEM dark field image, (B, H) an combined EDX maps of multiple elements (Cu in green, Zn in red and Zr in magenta) and (C-F, I-L) the individual EDX maps of the indicated elements.

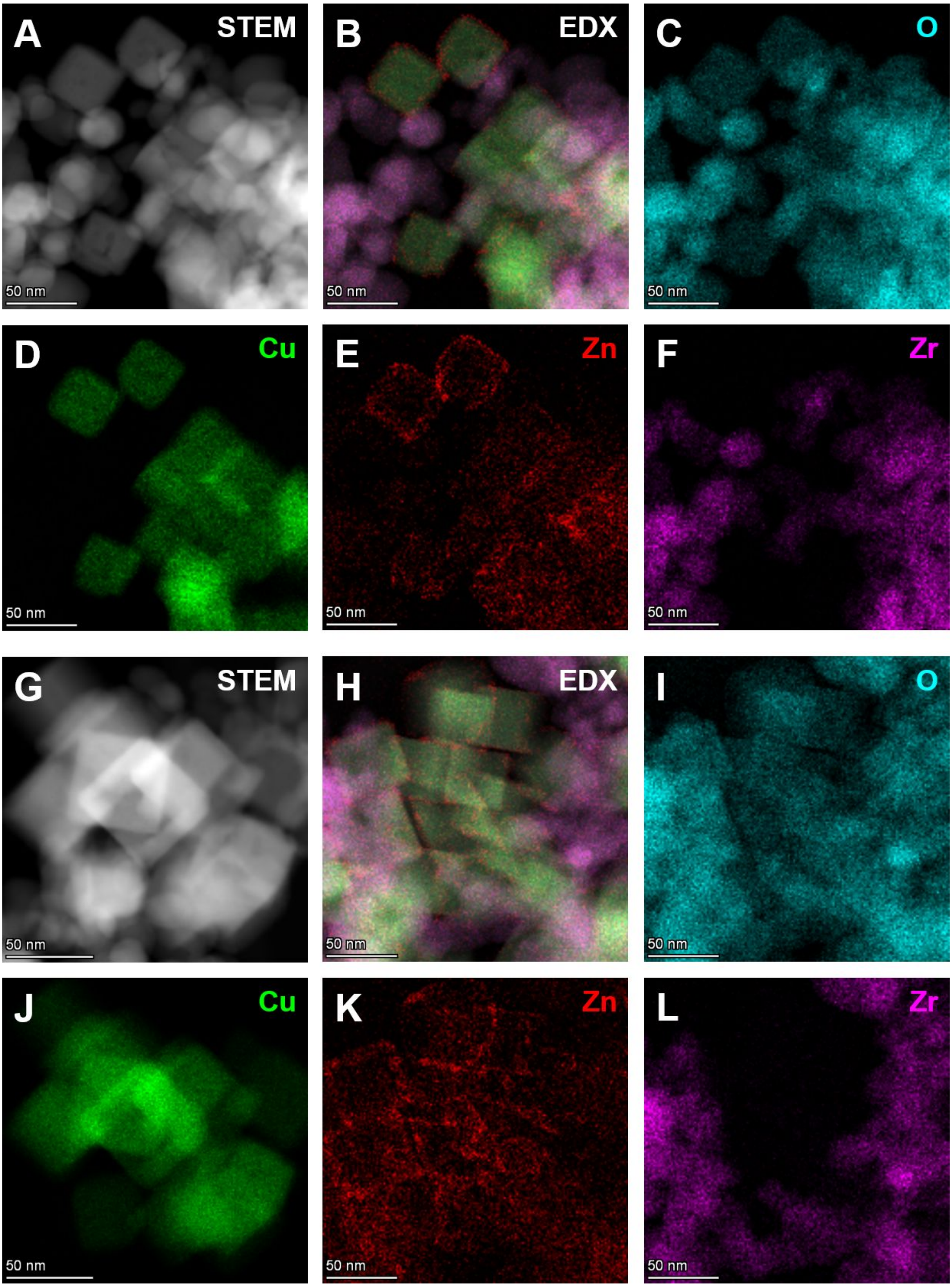

Fig. S10 STEM images and EDX maps of the as prepared ZnO/Cu_2O NCs with the high Zn loading supported on ZrO_2. Shown are (A, G) the STEM dark field image, (B, H) a combined EDX maps of multiple elements (Cu in green, Zn in red and Zr in magenta) and (C-F, I-L) the individual EDX maps of the indicated elements.

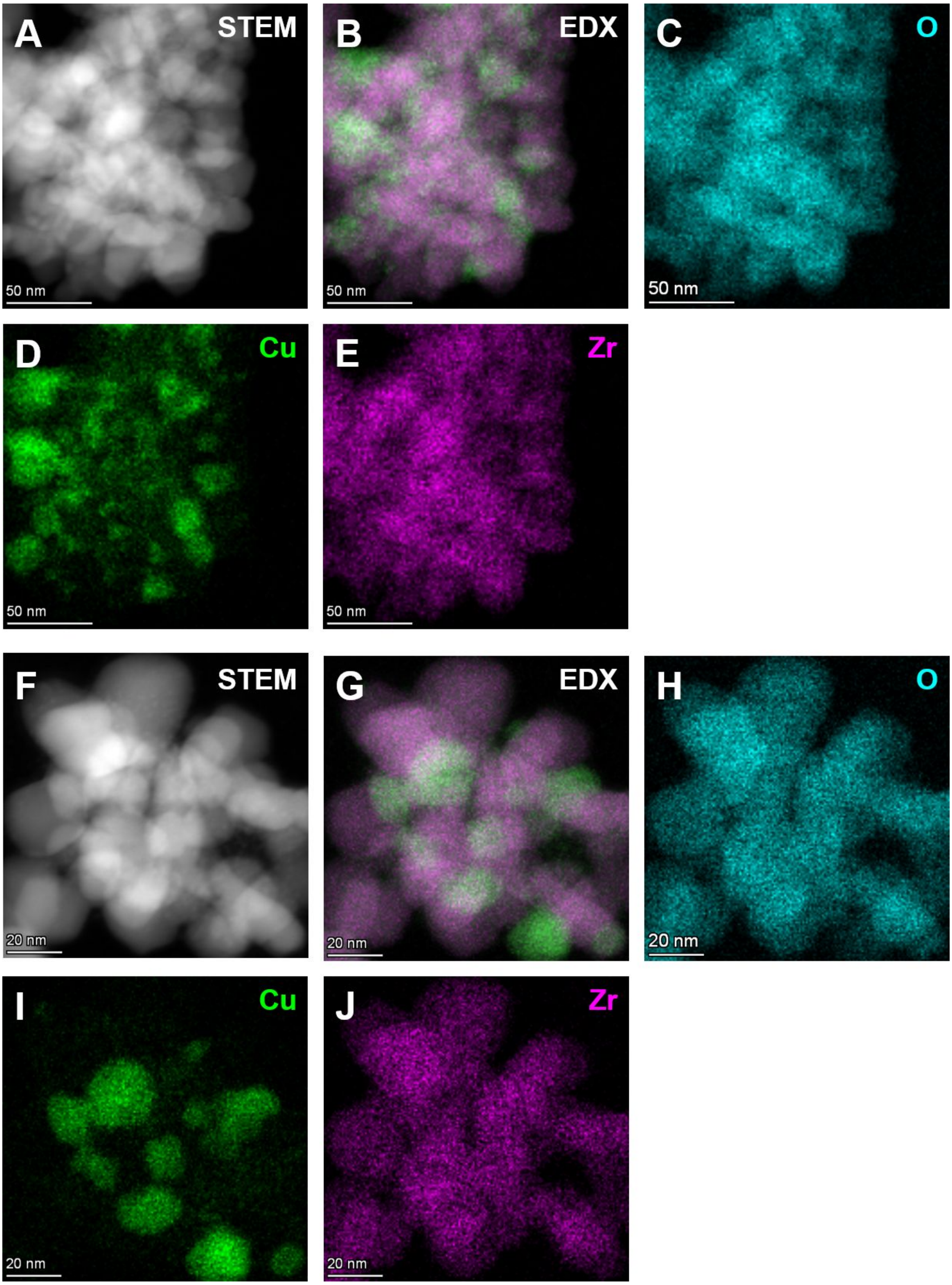

Fig. S11 STEM images and EDX maps of the Cu_2O NCs supported on ZrO_2 after reaction. Shown are (A, F) the STEM dark field image, (B, G) a combined EDX maps of multiple elements (Cu in green and Zr in magenta) and (C-E, H-J) the individual EDX maps of the indicated elements.

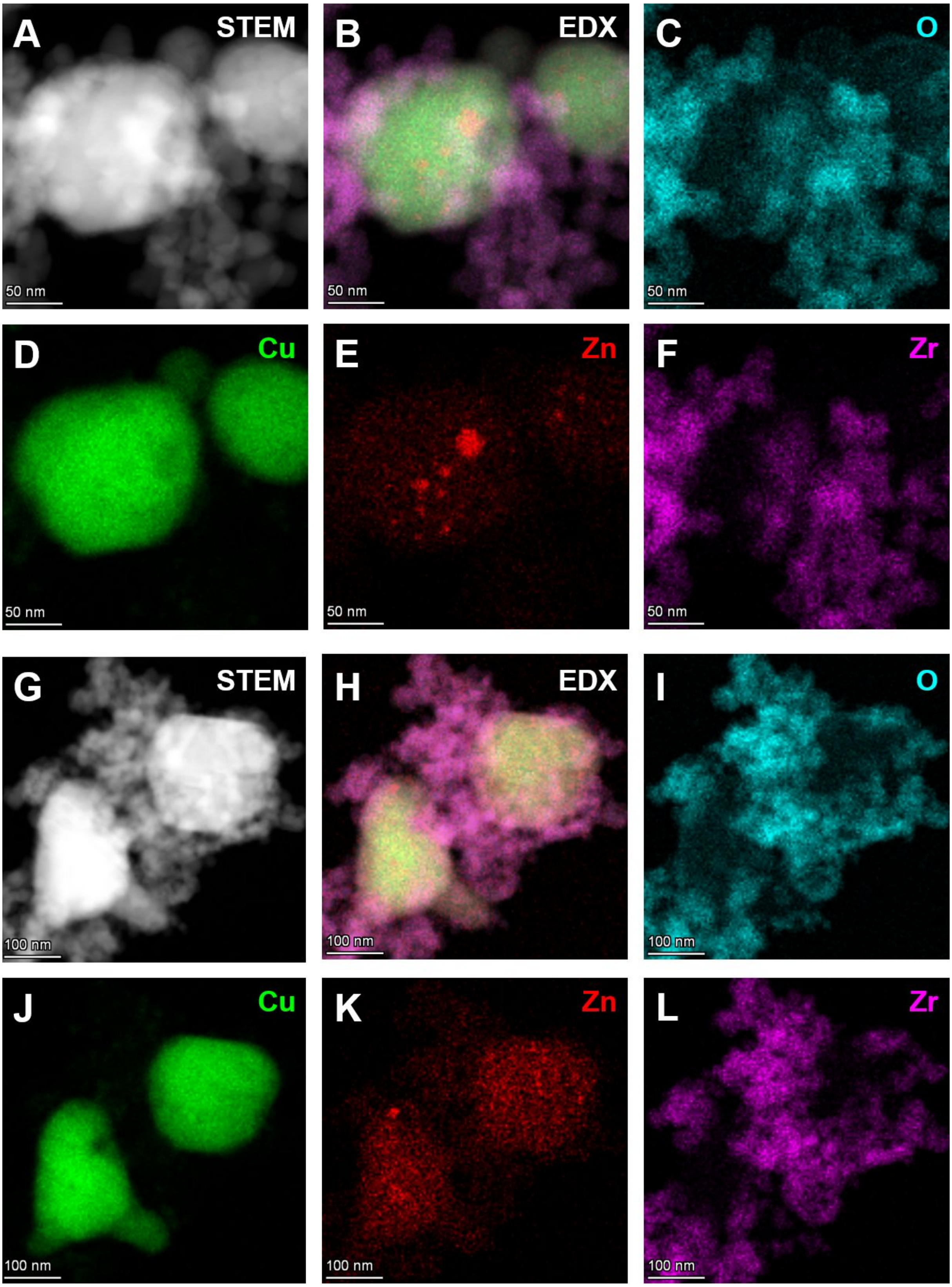

Fig. S12 STEM images and EDX maps of the ZnO/Cu_2O NCs with the low Zn loading supported on ZrO_2 after reaction. Shown are (A, G) the STEM dark field image, (B, H) an combined EDX maps of multiple elements (Cu in green, Zn in red and Zr in magenta) and (C-F, I-L) the individual EDX maps of the indicated elements.

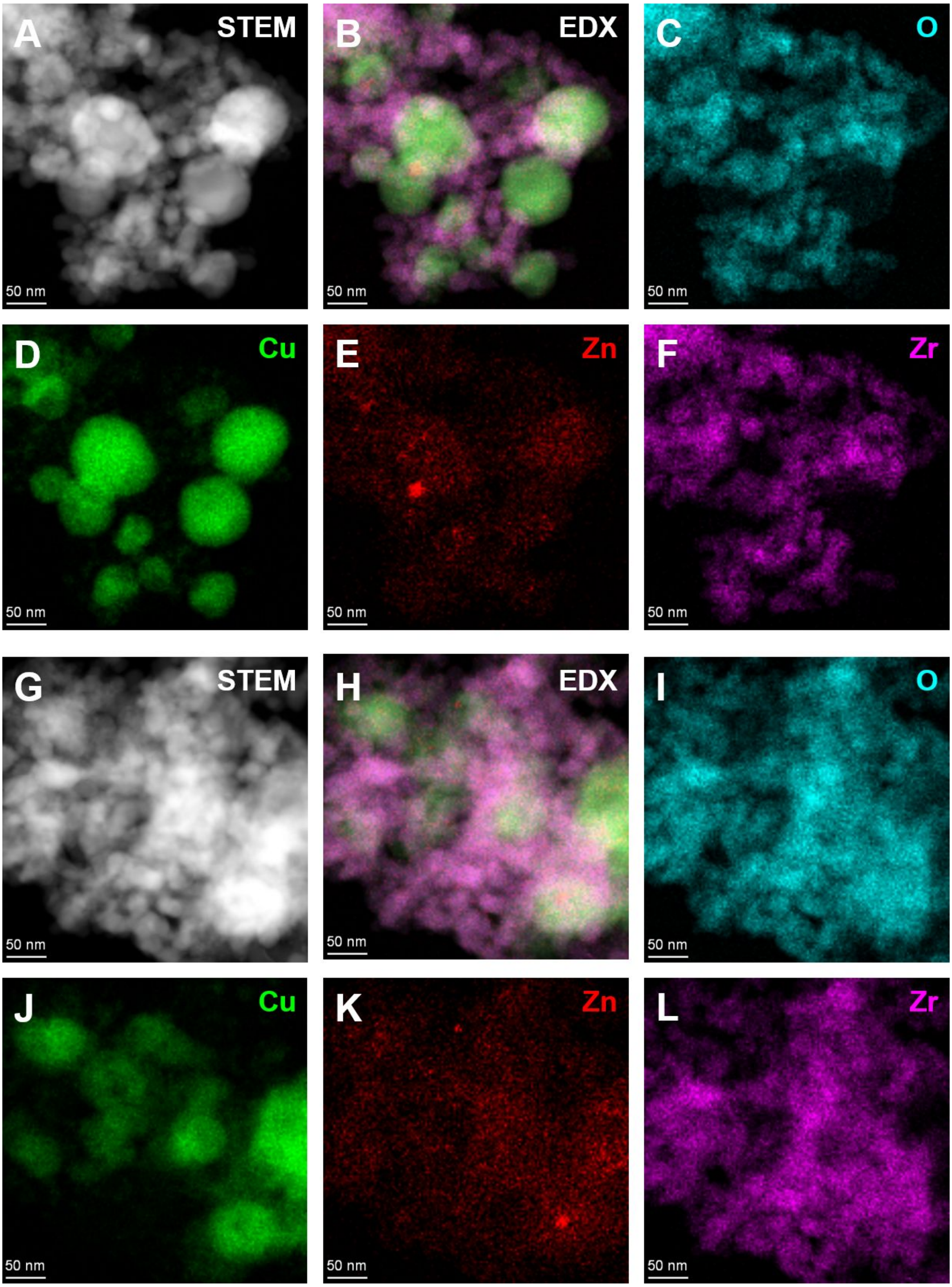

Fig. S13 STEM images and EDX maps of the ZnO/Cu_2O NCs with the medium Zn loading supported on ZrO_2 after reaction. Shown are (A, G) the STEM dark field image, (B, H) an combined EDX maps of multiple elements (Cu in green, Zn in red and Zr in magenta) and (C-F, I-L) the individual EDX maps of the indicated elements.

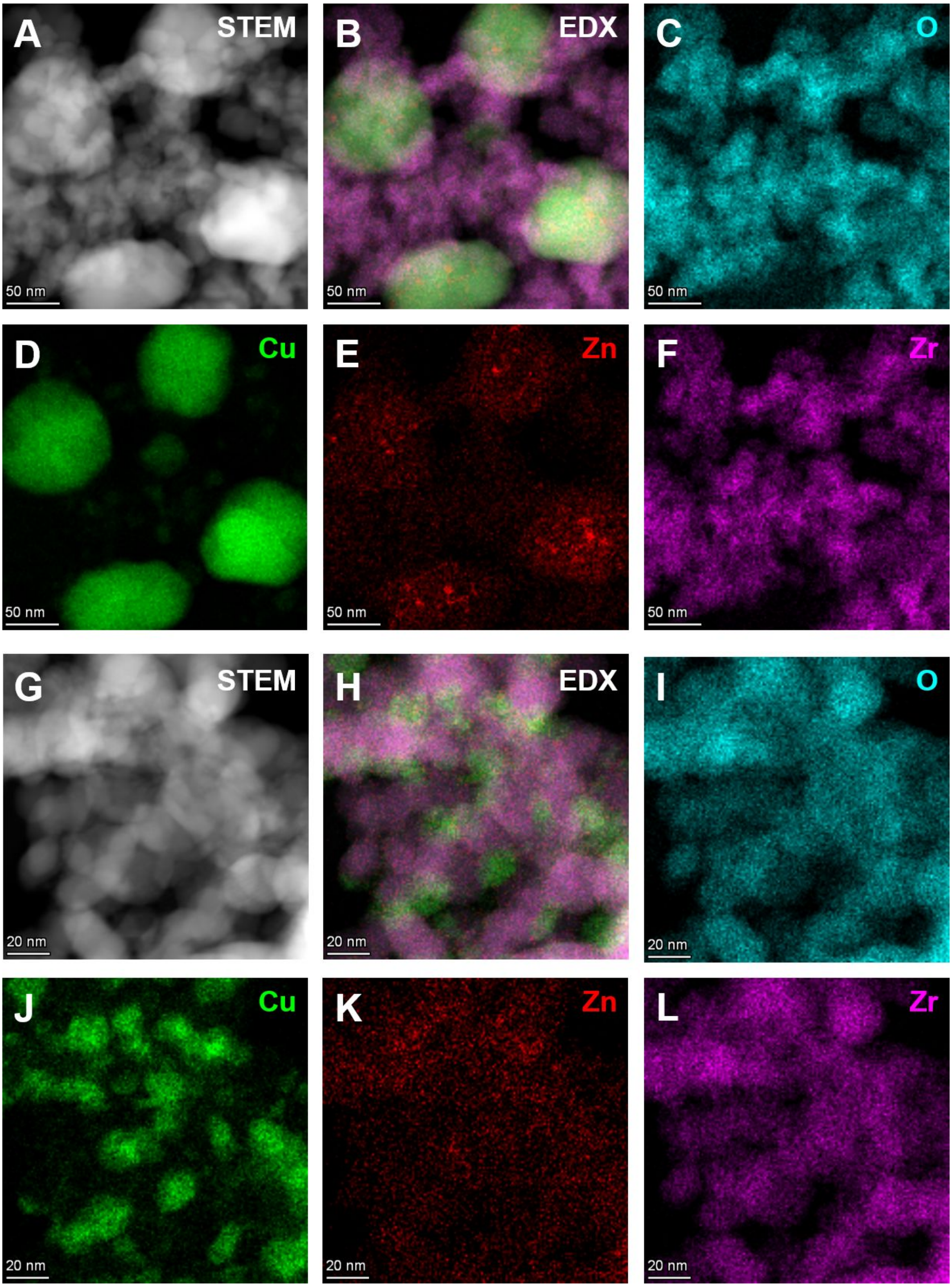

Fig. S14 STEM images and EDX maps of the ZnO/Cu_2O NCs with the high Zn loading supported on ZrO_2 after reaction. Shown are (A, G) the STEM dark field image, (B, H) an combined EDX maps of multiple elements (Cu in green, Zn in red and Zr in magenta) and (C-F, I-L) the individual EDX maps of the indicated elements.

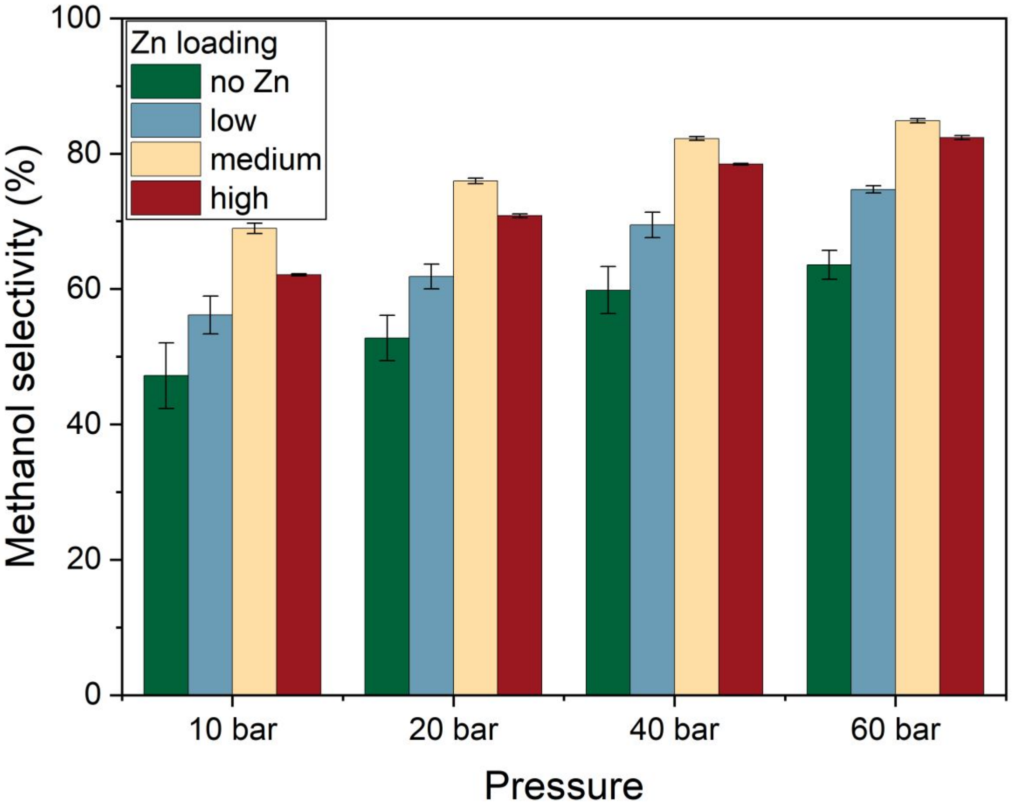

Fig. S15 Selectivity towards methanol of ZnO/Cu_2O NC catalysts on ZrO_2 with different Zn loadings. Apart from methanol, only CO and CH_4 were produced. The products not displayed here are mainly comprised of CO. Only minor amounts of CH_4 (< 0.4%) were detected. Measurements were performed under a $H_2 + CO_2 + He$ (3:1:1) atmosphere at various pressures and a temperature of 250 °C.

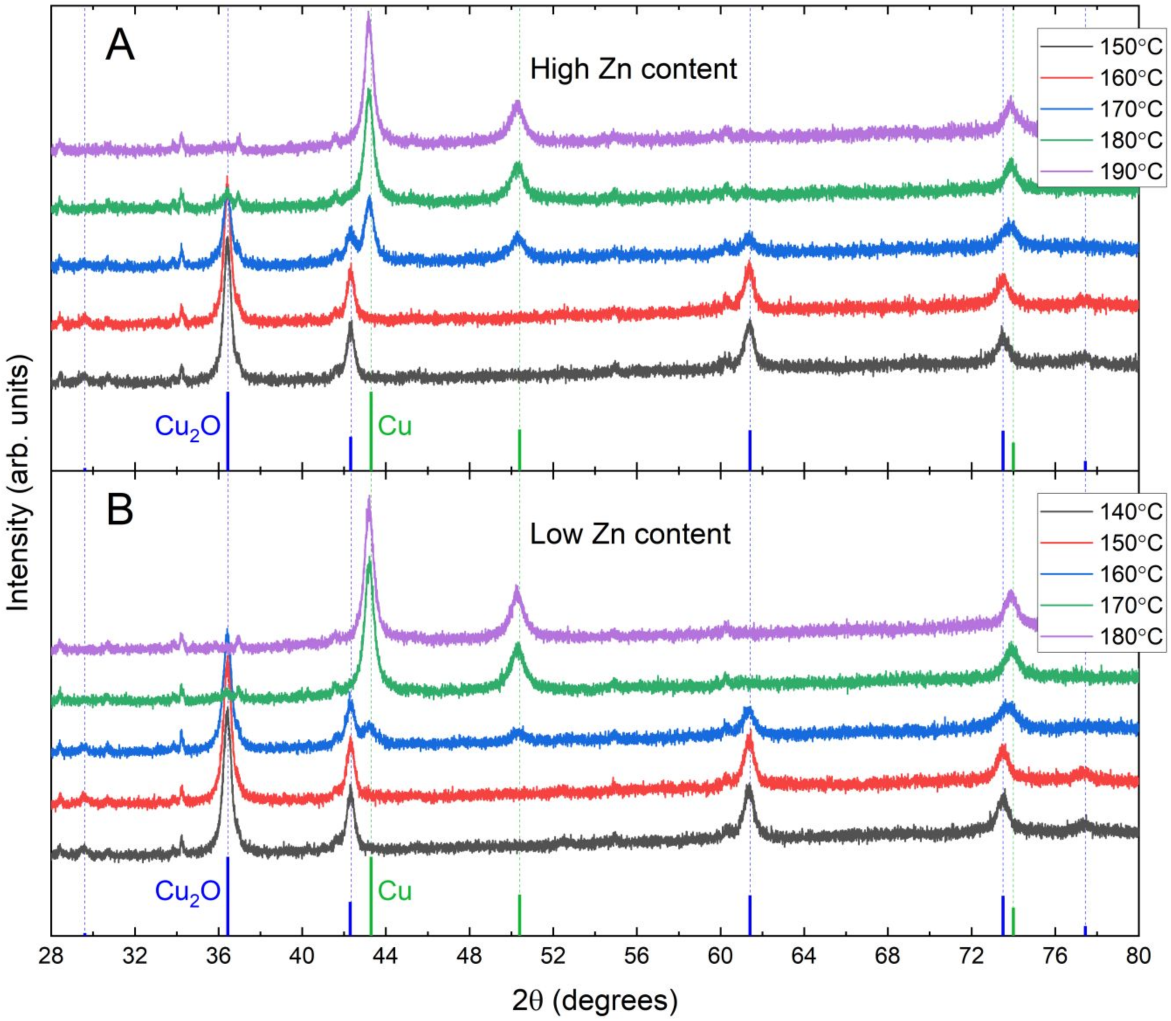

Fig. S16 XRD patterns corresponding to *in situ* XRD measurements during the reduction of the catalysts with high and low Zn content at selected temperatures. The reduction was done in 10% H_2 diluted with He and in 10 °C steps from 100 – 250 °C. The selected spectra show the transition from Cu_2O to metallic Cu. The peaks in the diffractogram that are not assigned by the reference vertical bars originate from the background of the sample holder.

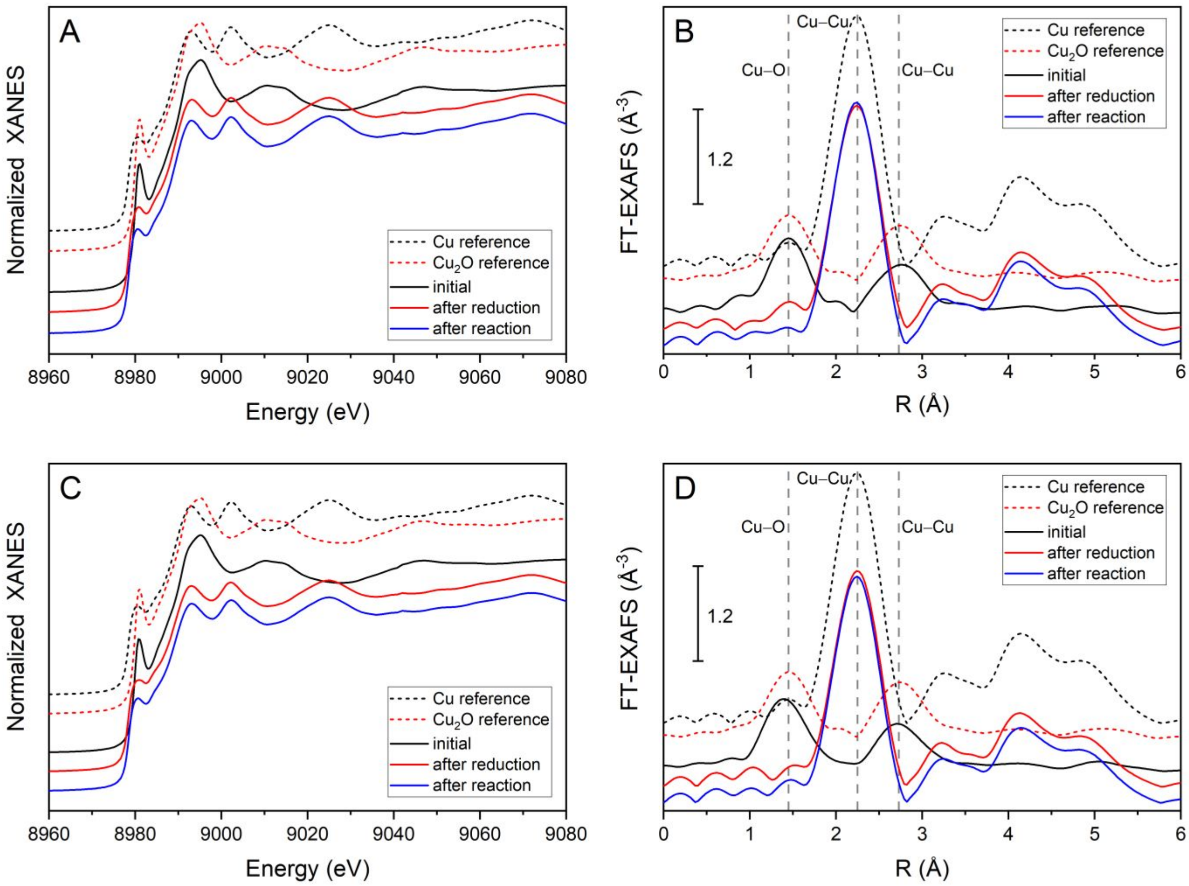

Fig. S17 *Operando* XAS spectra of the Cu K-edge of Cu_2O cubes with a ZnO shell supported on SiO_2. Shown are the (A) XANES and (B) EXAFS spectra for a high loading of Zn on the particles in the initial state, after the reduction (245 °C, 10% H_2 in He) and after the reaction treatments (250 °C, 75% H_2 + 25% CO_2, 20 bar). These measurements were performed after cooling down the samples (< 50 °C) to avoid contributions from thermal disorder. The same way (C) XANES and (D) EXAFS spectra are presented for a catalyst with low Zn loading. Additionally, reference spectra for bulk metallic Cu and Cu_2O are shown. The dashed lines indicate the peak positions of the Cu—O (1.45 Å) and Cu—Cu (2.73 Å) bonds of Cu_2O and the Cu—Cu bonds (2.25 Å) of metallic Cu, which were obtained from the reference materials.

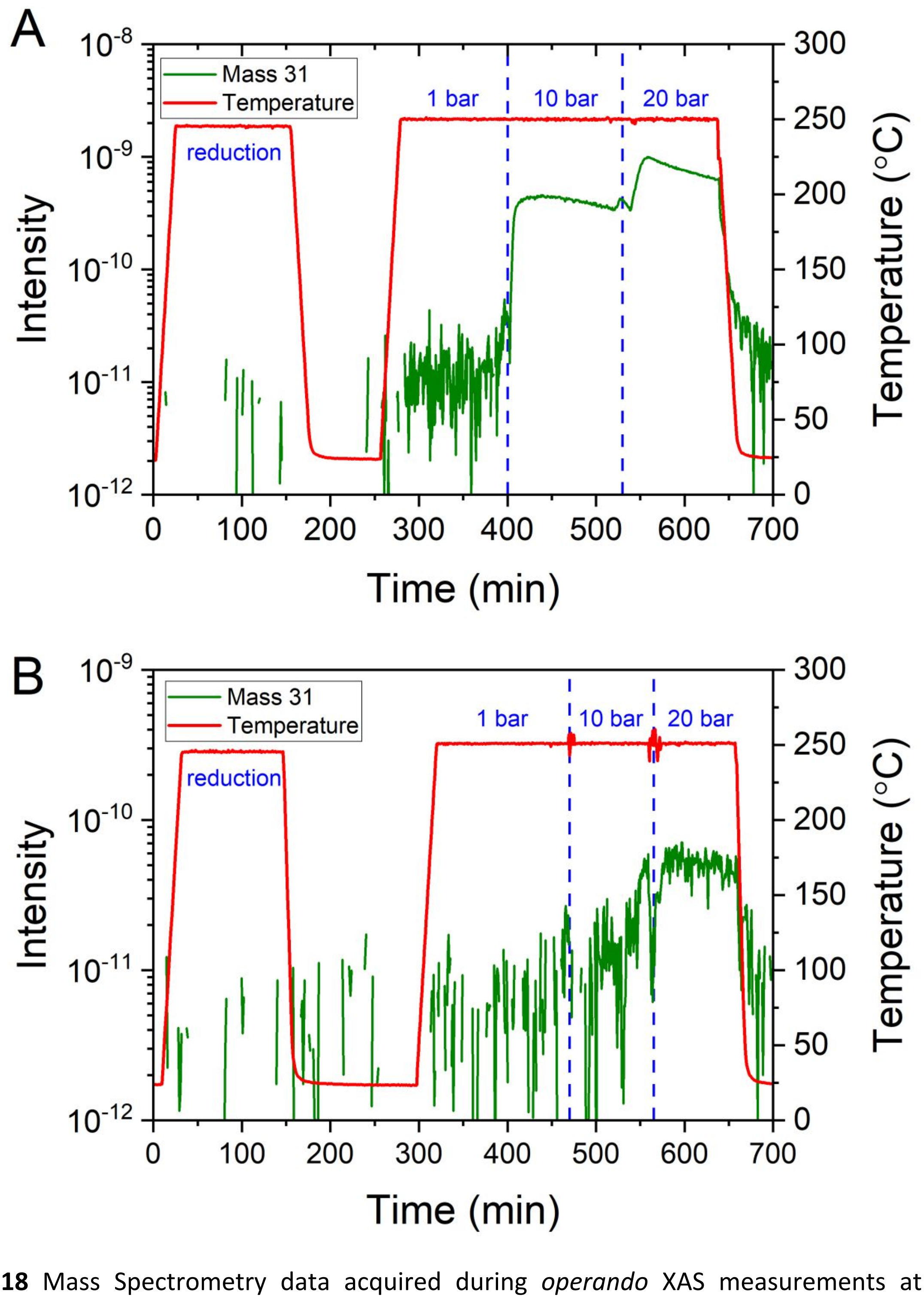

Fig. S18 Mass Spectrometry data acquired during *operando* XAS measurements at the synchrotron for ZnO/Cu_2O NC catalysts deposited on SiO_2 with (A) high and (B) low Zn loading. Shown is mass 31 corresponding to methanol, which is produced during the reaction. Significant amounts of methanol above the noise level can only be observed at high pressures (10 bar, 20 bar).

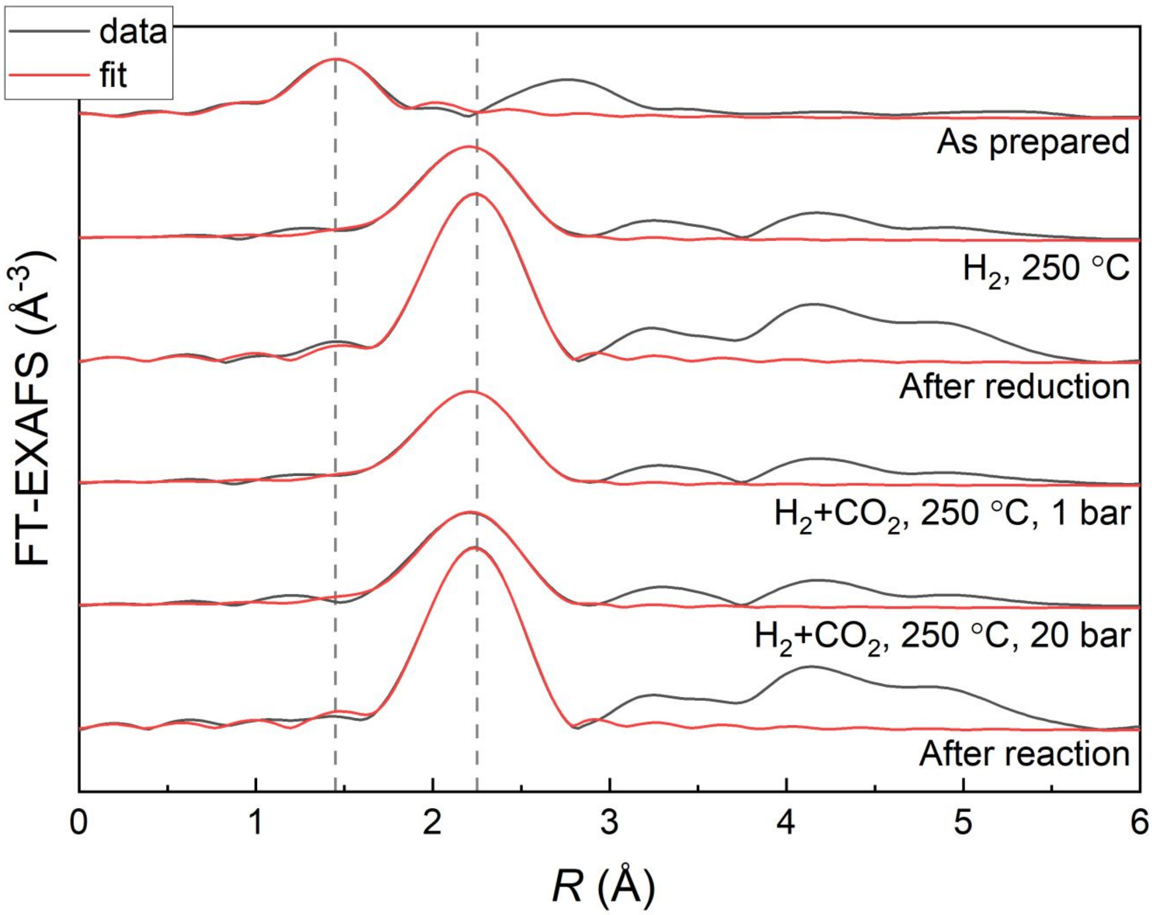

Fig. S19 Fitted FT-EXAFS spectra of the Cu K-edge for the ZnO/Cu_2O NC catalyst with the high loading of Zn deposited on SiO_2. The reference lines indicate the positions of the first shell Cu-O bonds (for Cu_2O) and Cu-Cu (for metallic Cu) bonds, which were obtained from reference materials.

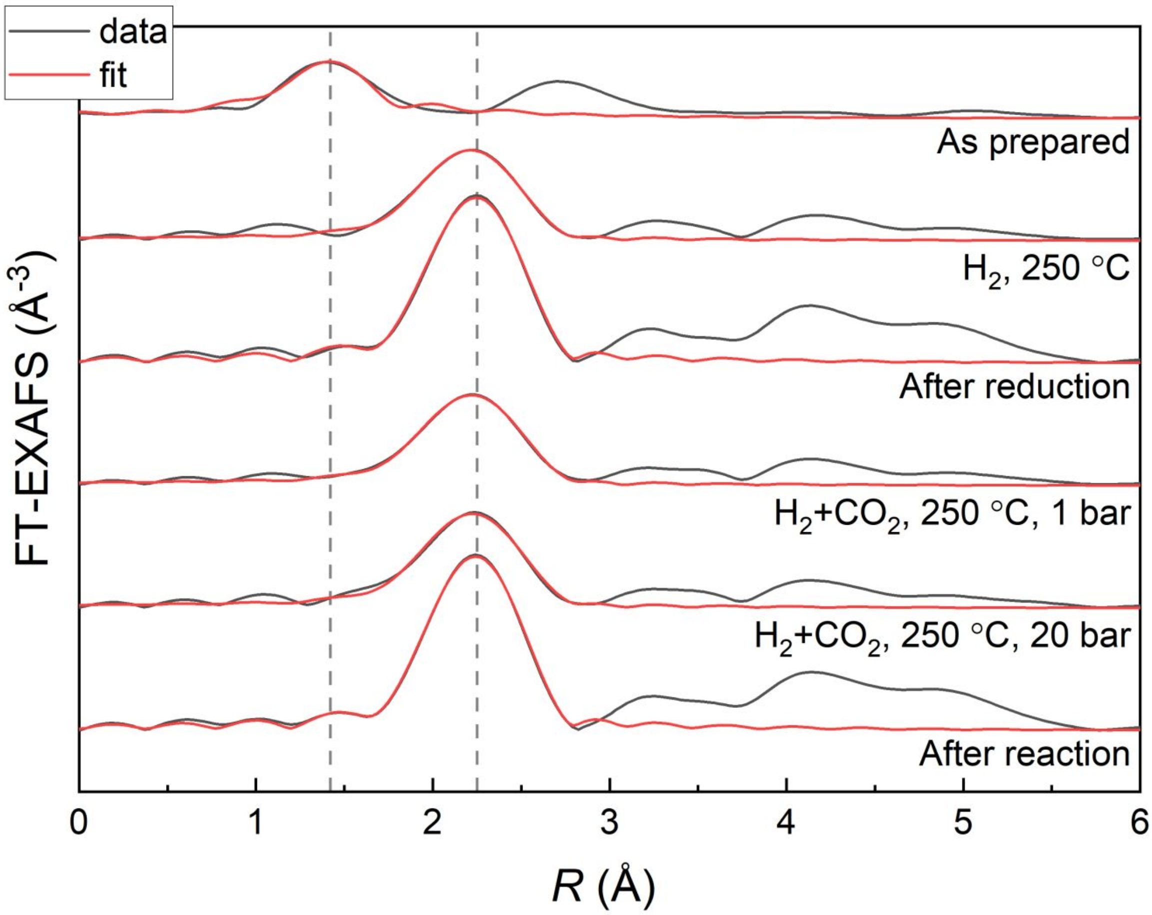

Fig. S20 Fitted FT-EXAFS spectra of the Cu K-edge for the ZnO/Cu_2O NC catalyst with the low loading of Zn deposited on SiO_2. The reference lines indicate the positions of the first shell Cu-O bonds (for Cu_2O) and Cu-Cu (for metallic Cu) bonds, which were obtained from reference materials.

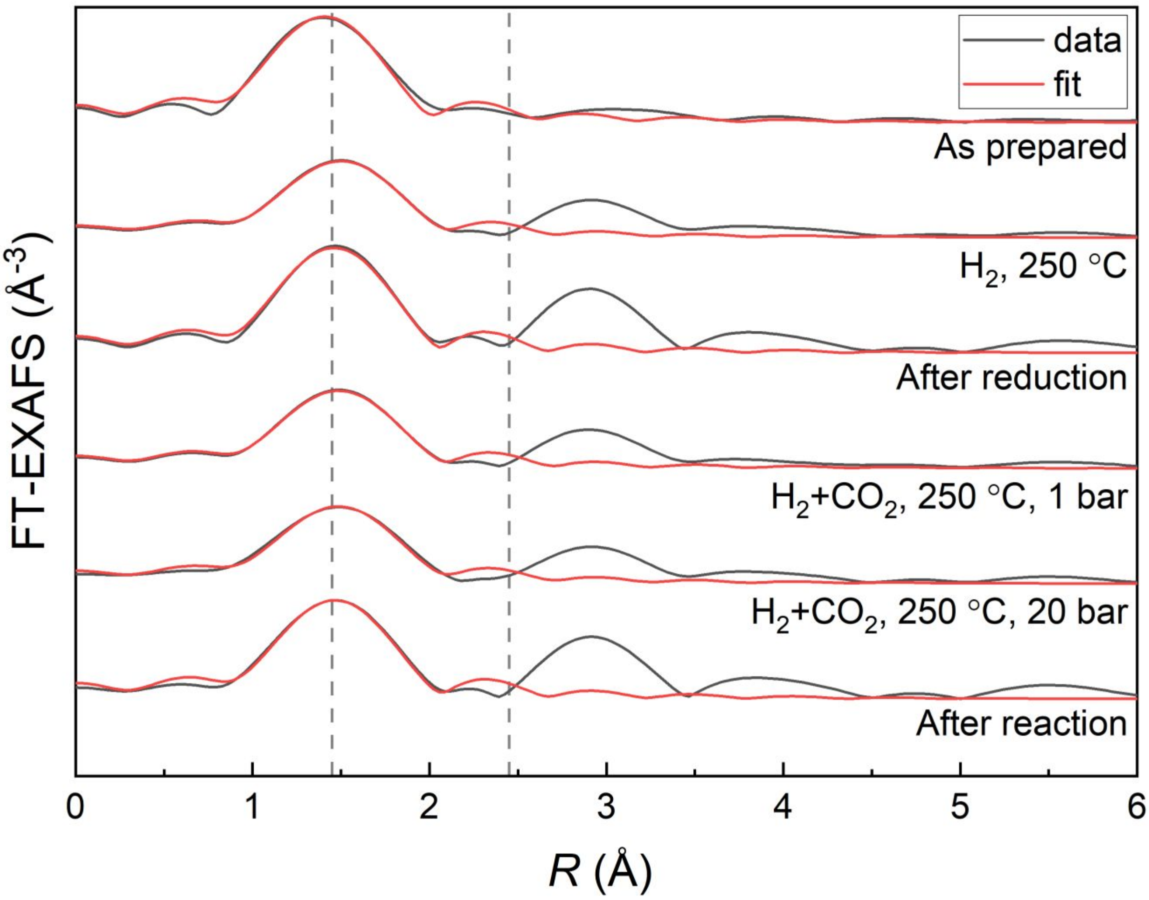

Fig. S21 Fitted FT-EXAFS spectra of the Zn K-edge for the ZnO/Cu_2O NC with the high loading of Zn catalyst deposited on SiO_2. The reference lines indicate the positions of the first shell Zn-O bonds (for ZnO) and Zn-Zn (for metallic Zn) bonds, which were obtained from reference materials.

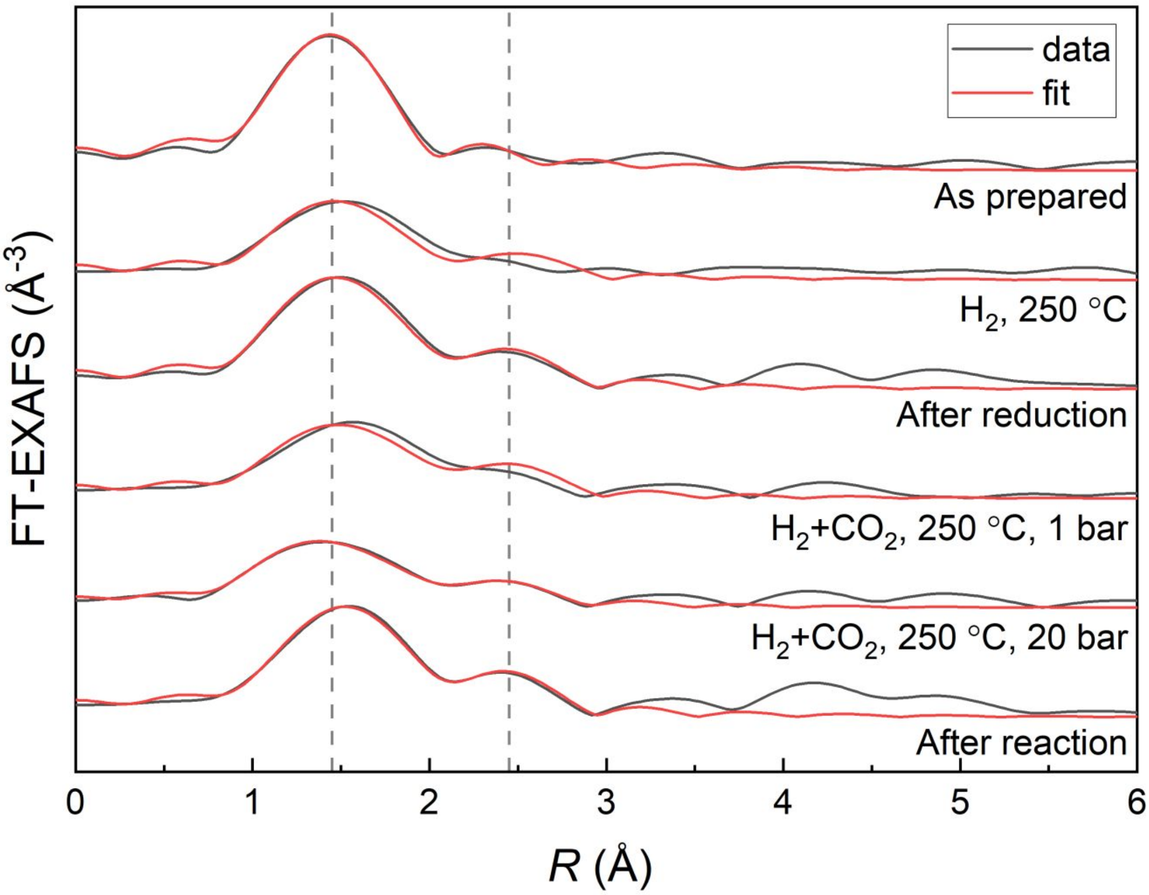

Fig. S22 Fitted FT-EXAFS spectra of the Zn K-edge for the ZnO/Cu_2O catalysts with the low loading of Zn deposited on SiO_2. The reference lines indicate the positions of the first shell Zn-O bonds (for ZnO) and Zn-Zn (for metallic Zn) bonds, which were obtained from reference materials.

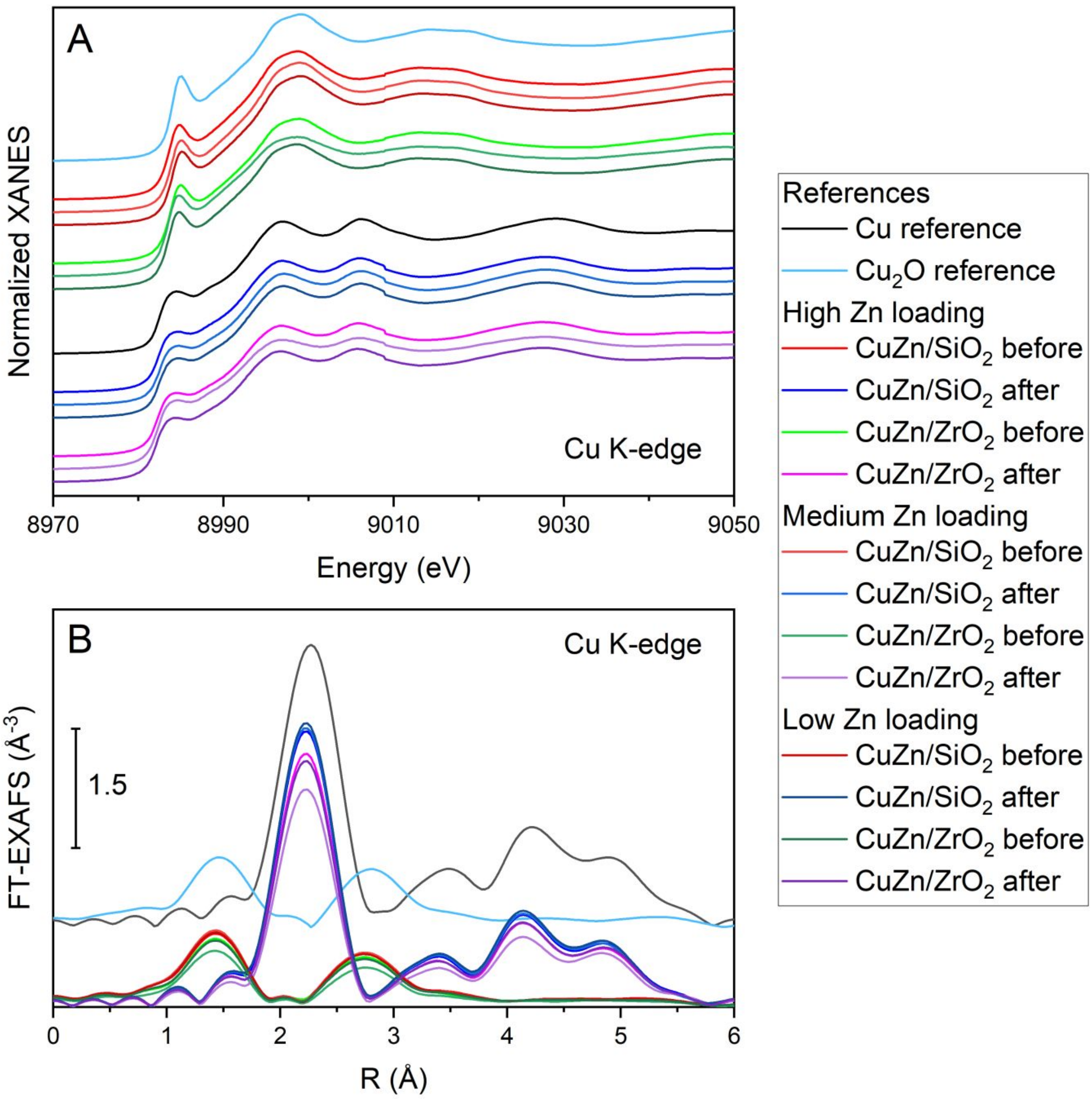

Fig. S23 (A) Normalized XANES and (B) Fourier-transformed EXAFS spectra of SiO_2 and ZrO_2 - supported ZnO/Cu_2O NC samples at the Cu K-edge. Spectra were acquired before and after reduction (H_2, p = 1 bar, T = 245 °C). Additionally, Cu and Cu_2O reference spectra are shown. The initial state of all samples is predominantly Cu_2O, while after the treatment it is metallic Cu.

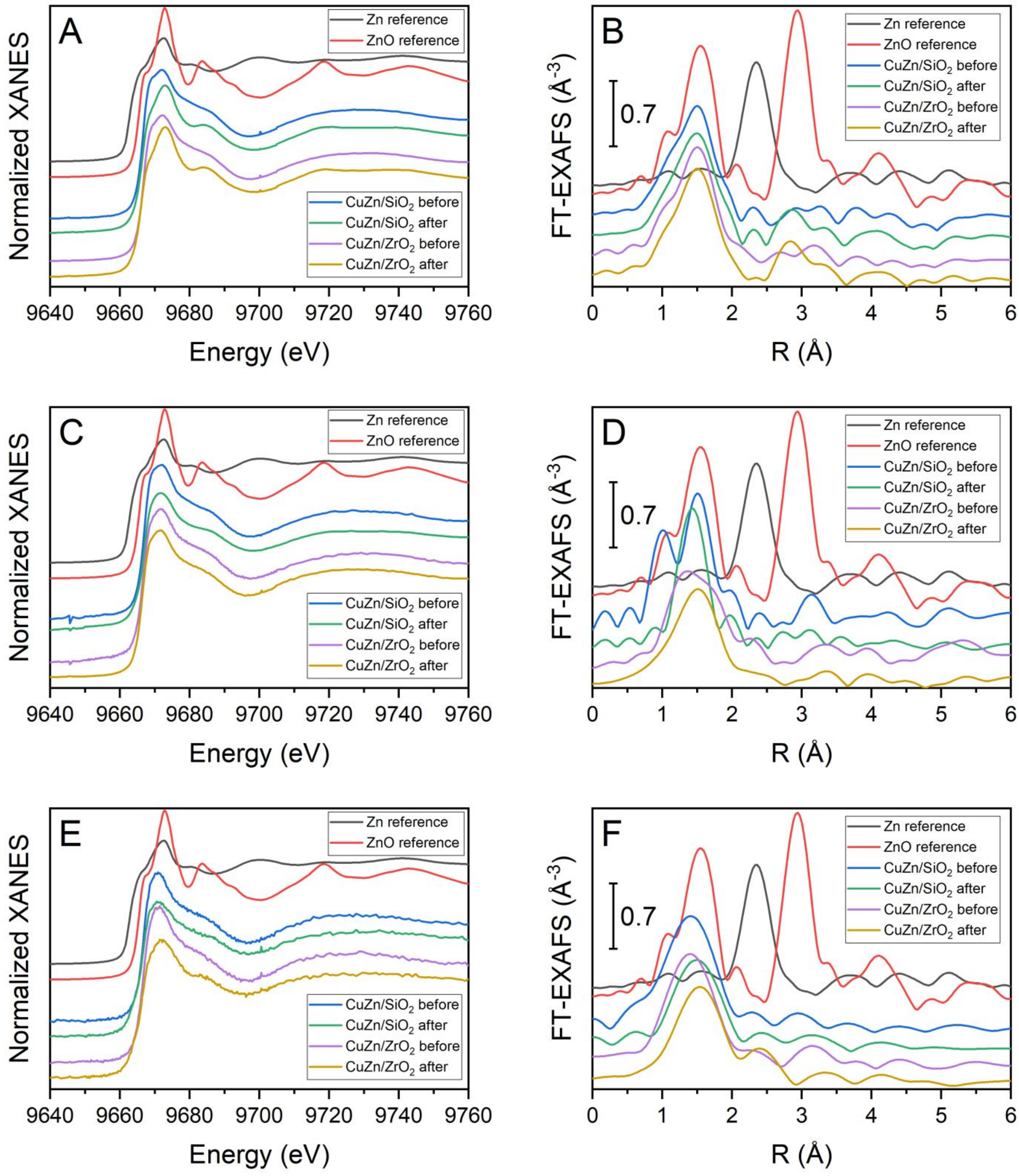

Fig. S24 Normalized XANES and Fourier-transformed EXAFS spectra of SiO_2 and ZrO_2-supported Cu_2O/ZnO samples at the Zn K-edge. Shown are the XANES and corresponding EXAFS spectra for the catalysts with a (A, B) high, (C, D) intermediate and (E, F) low loading of Zn on the catalyst. Spectra were acquired before and after reduction (H_2, p = 1 bar, T = 245 °C). Additionally, reference spectra for bulk metallic Zn and ZnO are shown.

Sample	Phase	Space group	*X* / wt%	*a* / Å	*c* / Å
High Zn, initial state	Cu_2O	$Pn\bar{3}m$	100	4.274 (4)	-
Low Zn, initial state	Cu_2O	$Pn\bar{3}m$	100	4.273 (5)	-
High Zn, after reaction	Cu	$Fm\bar{3}m$	94.8	3.634 (3)	-
	ZnO	$P6_3mc$	5.2	3.253 (4)	5.231 (5)
Low Zn, after reaction	Cu	$Fm\bar{3}m$	100	3.621 (4)	-
Cu reference	Cu	$Fm\bar{3}m$	-	3.615 (12)	-
ZnO reference	ZnO	$P6_3mc$	-	3.2496 (3)	5.2058 (4)

Table. S2 XRD fitting parameters extracted from Rietveld refinement. No crystalline Zn species were detected in all ZnO/Cu_2O catalysts, except for the catalyst with a high Zn loading after reaction. Parameters for Cu and ZnO reference samples were acquired in the same configuration. The corresponding spectra for the references are shown in Fig. S1.

Temperature (°C)	Cu lattice constant *a* / Å	
	High Zn loading	Low Zn loading
220	3.631 (4)	3.634 (4)
250	3.634 (5)	3.636 (4)
400	3.661 (4)	3.647 (4)
250	3.647 (4)	3.635 (4)
220	3.646 (3)	3.632 (4)

Table. S3 Cu lattice constant extracted from XRD measurements at different reaction temperatures in $H_2 + CO_2$ at 10 bar.

Condition	CN	ΔR	σ^2	ΔE_0	R-factor
**Low Zn loading (Cu-O)**					
Initial state	4.7 (1.2)	-0.11 (2)	0.003 (3)	5 (3)	0.03
**Low Zn loading (Cu-M, M=Cu,Zn)**					
Initial state	-	-	-	-	-
Reduction (H_2, 245 °C)	10.0 (1.1)	-0.021 (8)	0.0148 (12)	3.4 (1.0)	0.07
After reduction	10.2 (0.4)	-0.008 (3)	0.0086 (4)	5.0 (0.4)	0.0009
Reaction (250 °C, 1 bar)	10.6 (0.7)	-0.017 (4)	0.0152 (7)	3.8 (0.6)	0.002
Reaction (250 °C, 20 bar)	10.7 (1.1)	-0.016 (7)	0.0149 (11)	4.0 (0.9)	0.006
After reaction	10.6 (0.3)	-0.011 (2)	0.0086 (3)	4.9 (0.3)	0.0004
**High Zn loading (Cu-O)**					
Initial state	4.0 (0.6)	-0.09 (1)	0.0008 (20)	8 (2)	0.012
**High Zn loading (Cu-M, M=Cu,Zn)**					
Initial state	-	-	-	-	-
Reduction (H_2, 245 °C)	11.6 (1.0)	-0.024 (6)	0.0158 (10)	3.1 (0.8)	0.004
After reduction	10.9 (0.5)	-0.011 (3)	0.0091 (4)	4.8 (0.5)	0.0012
Reaction (250 °C, 1 bar)	11.6 (0.8)	-0.021 (5)	0.0158 (8)	3.4 (0.6)	0.002
Reaction (250 °C, 20 bar)	11.4 (1.4)	-0.022 (8)	0.0154 (13)	3.5 (1.1)	0.008
After reaction	11.5 (0.6)	-0.013 (4)	0.0089 (5)	4.4 (0.6)	0.002

Tab. S4 EXAFS first-shell fitting parameters for Cu K-edge of samples measured in the flow reactor at SSRL synchrotron (USA). Initial state was acquired before any treatment in a He atmosphere at room temperature. Parameters for the after reduction/reaction were acquired from scans performed after cooling sample down close to room temperature (<50°C).

Condition	CN	ΔR	σ^2	ΔE_0	R-factor
**Low Zn loading (Zn-O)**					
Initial state	4.0 (0.4)	0.16 (1)	0.004 (2)	3 (1)	0.005
Reduction (H_2, 245 °C)	1.7 (3.0)	0.14 (17)	0.00 (3)	4 (19)	0.027
After reduction	2.5 (0.6)	0.13 (2)	0.000 (4)	2 (2)	0.004
Reaction (250 °C, 1 bar)	1.6 (1.0)	0.13(6)	0.00 (1)	3 (6)	0.025
Reaction (250 °C, 20 bar)	1.7 (0.4)	0.08 (2)	0.000 (4)	-2 (3)	0.003
After reaction	2.5 (0.4)	0.15 (2)	0.0001 (29)	3 (2)	0.002
**Low Zn loading (Zn-M, M=Cu,Zn)**					
Initial state	-	-	-	-	-
Reduction (H_2, 245 °C)	3 (18)	-0.06 (24)	0.01 (8)	4 (19)	0.027
After reduction	2.2 (1.8)	0.09 (2)	0.006 (10)	2 (2)	0.004
Reaction (250 °C, 1 bar)	2.5 (3.8)	-0.08 (6)	0.009 (19)	3 (6)	0.025
Reaction (250 °C, 20 bar)	1.8 (1.1)	-0.12 (3)	0.007 (7)	-2 (3)	0.003
After reaction	2.4 (1.3)	-0.08 (2)	0.006 (7)	3 (2)	0.002
**High Zn loading (Zn-O)**					
Initial state	4.2 (0.8)	0.14 (2)	0.002 (3)	0.7 (2.2)	0.013
Reduction (H_2, 245 °C)	3.0 (0.5)	0.18 (2)	0.002 (3)	6 (2)	0.007
After reduction	3.6 (0.8)	0.16 (2)	0.000 (3)	4 (2)	0.011
Reaction (250 °C, 1 bar)	2.9 (0.5)	0.18 (2)	0.001 (3)	6 (2)	0.007
Reaction (250 °C, 20 bar)	3.3 (0.8)	0.18 (2)	0.004 (4)	6 (2)	0.015
After reaction	3.5 (0.7)	0.17 (2)	0.0007 (33)	4 (2)	0.010

Tab. S5 EXAFS fit parameters for the Zn K-edge of samples measured in the flow reactor at the SSRL synchrotron (USA). Initial state was acquired before any treatment in a He atmosphere at room temperature. Parameters for the after reduction/reaction were acquired from scans performed after cooling sample down close to room temperature (<50°C).

Supplementary References

1. Simonelli, L. *et al.* CLÆSS: The hard X-ray absorption beamline of the ALBA CELLS synchrotron. *Cogent Phys.* **3**, 1231987 (2016).
2. Cumpson, P. J. & Zalm, C. The Thickogram: a method for easy film thickness measurement in XPS. *Surf. Interface Anal* **29**, 403–406 (2000).
3. Tanuma, S., Powell, C. J. & Penn, D. R. Calculations of electron inelastic mean free paths. V. Data for 14 organic compounds over the 50–2000 eV range. *Surf. Interface Anal.* **21**, 165–176 (1994).
4. Behrens, M. *et al.* The active site of methanol synthesis over $Cu/ZnO/Al_2O_3$ industrial catalysts. *Science* **336**, 893–897 (2012).
5. Sehested, J. Industrial and scientific directions of methanol catalyst development. *Journal of Catalysis* **371**, 368–375 (2019).
6. Kuld, S., Conradsen, C., Moses, P. G., Chorkendorff, I. & Sehested, J. Quantification of zinc atoms in a surface alloy on copper in an industrial-type methanol synthesis catalyst. *Angew. Chemie - Int. Ed.* **53**, 5941–5945 (2014).
7. Muhler, M., Törnqvist, E., Nielsen, L. P., Clausen, B. S. & Topsøe, H. On the role of adsorbed atomic oxygen and CO_2 in copper based methanol synthesis catalysts. *Catal. Letters* **25**, 1–10 (1994).
8. Studt, F. *et al.* The Mechanism of CO and CO_2 Hydrogenation to Methanol over Cu-Based Catalysts. *ChemCatChem* **7**, 1105–1111 (2015).
9. Chinchen, G. C., Denny, P. J., Parker, D. G., Spencer, M. S. & Whan, D. A. Mechanism of methanol synthesis from $CO_2/CO/H_2$ mixtures over copper/zinc oxide/alumina catalysts: use of 14C-labelled reactants. *Appl. Catal.* **30**, 333–338 (1987).
10. Nielsen, N. D., Jensen, A. D. & Christensen, J. M. The roles of CO and CO_2 in high pressure methanol synthesis over Cu-based catalysts. *J. Catal.* **393**, 324–334 (2021).
11. Li, K. & Chen, J. G. CO_2 Hydrogenation to Methanol over ZrO_2-Containing Catalysts: Insights into ZrO_2 Induced Synergy. *ACS Catal.* **9**, 7840–7861 (2019).
12. Denise, B. & Sneeden, R. P. A. Oxide-supported copper catalysts prepared from copper formate: Differences in behavior in methanol synthesis from CO/H_2 and CO_2/H_2 mixtures. *Appl. Catal.* **28**, 235-239 (1986).
13. Amenomiya, Y. Methanol synthesis from CO_2 + H_2 II. Copper-based binary and ternary catalysts. *Appl. Catal.* **30**, 57-68 (1987).
14. Arena, F. *et al.* Synthesis, characterization and activity pattern of $Cu\text{-}ZnO/ZrO_2$ catalysts in the hydrogenation of carbon dioxide to methanol. *J. Catal.* **249**, 185–194 (2007).
15. Fujitani, T. *et al.* The role of metal oxides in promoting a copper catalyst for methanol synthesis. *Catal. Letters* **25**, 271–276 (1994).
16. Saito, M. *et al.* Development of Cu/ZnO-based high performance catalysts for methanol synthesis by CO_2 hydrogenation. *Energy Convers. Manag.* **36**, 577–580 (1995).

17. Wang, L. C. *et al.* Structural evolution and catalytic properties of nanostructured Cu/ZrO_2 catalysts prepared by oxalate gel-coprecipitation technique. *J. Phys. Chem. C* **111**, 16549–16557 (2007).

18. Tada, S. *et al.* Design of Interfacial Sites between Cu and Amorphous ZrO_2 Dedicated to CO_2-to-Methanol Hydrogenation. *ACS Catal.* **8**, 7809–7819 (2018).

19. Nitta, Y., Fujimatsu, T., Okamoto, Y. & Imanaka, T. Effect of starting salt on catalytic behaviour of Cu-ZrO_2 catalysts in methanol synthesis from carbon dioxide. *Catal. Letters* **17**, 157–165 (1993).

20. Samson, K. *et al.* Influence of ZrO_2 structure and copper electronic state on activity of Cu/ZrO_2 catalysts in methanol synthesis from CO_2. *ACS Catal.* **4**, 3730–3741 (2014).

21. Huang, C., Chen, S., Fei, X., Liu, D. & Zhang, Y. Catalytic hydrogenation of CO_2 to methanol: Study of synergistic effect on adsorption properties of CO_2 and H_2 in CuO/ZnO/ZrO_2 system. *Catalysts* **5**, 1846–1861 (2015).

22. Larmier, K. *et al.* CO_2-to-Methanol Hydrogenation on Zirconia-Supported Copper Nanoparticles: Reaction Intermediates and the Role of the Metal–Support Interface. *Angew. Chemie - Int. Ed.* **56**, 2318–2323 (2017).

23. Jung, K. T. & Bell, A. T. Effects of zirconia phase on the synthesis of methanol over zirconia-supported copper. *Catal. Letters* **80**, 63–68 (2002).

24. Ma, Z. Y., Yang, C., Wei, W., Li, W. H. & Sun, Y. H. Catalytic performance of copper supported on zirconia polymorphs for CO hydrogenation. *J. Mol. Catal. A Chem.* **231**, 75–81 (2005).

25. Witoon, T., Chalorngtham, J., Dumrongbunditkul, P., Chareonpanich, M. & Limtrakul, J. CO_2 hydrogenation to methanol over Cu/ZrO_2 catalysts: Effects of zirconia phases. *Chem. Eng. J.* **293**, 327–336 (2016).

26. Bonura, G., Cordaro, M., Cannilla, C., Arena, F. & Frusteri, F. The changing nature of the active site of Cu-Zn-Zr catalysts for the CO_2 hydrogenation reaction to methanol. *Appl. Catal. B Environ.* **152**–**153**, 152–161 (2014).

27. Wambach, J., Baiker, A. & Wokaun, A. CO_2 hydrogenation over metal/zirconia catalysts. *Phys. Chem. Chem. Phys.* **1**, 5071–5080 (1999).

28. Koeppel, R. A., Baiker, A. & Wokaun, A. Copper/zirconia catalysts for the synthesis of methanol from carbon dioxide. Influence of preparation variables on structural and catalytic properties of catalysts. *Appl. Catal. A, Gen.* **84**, 77–102 (1992).

29. Li, L., Mao, D., Yu, J. & Guo, X. Highly selective hydrogenation of CO_2 to methanol over CuO-ZnO-ZrO_2 catalysts prepared by a surfactant-assisted co-precipitation method. *J. Power Sources* **279**, 394–404 (2015).

30. Wang, Y. *et al.* Exploring the ternary interactions in Cu–ZnO–ZrO_2 catalysts for efficient CO_2 hydrogenation to methanol. *Nat. Commun.* **10**, (2019).
